# Nano-selenium enhances melon resistance to *Podosphaera xanthii* by enhancing the antioxidant capacity and promoting alterations in the polyamine, phenylpropanoid and hormone signaling pathways

**DOI:** 10.1186/s12951-023-02148-y

**Published:** 2023-10-16

**Authors:** Lu Kang, Yangliu Wu, Yujiao Jia, Zhendong Chen, Dexian Kang, Li Zhang, Canping Pan

**Affiliations:** 1 Key Laboratory of National Forestry and Grassland Administration on Pest Chemical Control & Innovation Center of Pesticide Research, College of Science, China Agricultural University, 2 Yuanmingyuan Western Road, Haidian District, Beijing, 100193 China; 2 Institute of Agricultural Quality Standards and Testing Technology, Xinjiang Academy of Agricultural Sciences, Urumqi, 830091 China; 3 School of Biological Science and Technology, University of Jinan, Jinan, 250022 China; 4 Vegetable Research Institute, Guangxi Zhuang Autonomous Region Academy of Agricultural Sciences, Nanning, 530000 China

**Keywords:** Melon, Powdery mildew, Nano-Se, Polyamine, Salicylic acid

## Abstract

Powdery mildew is one of the main problematic diseases in melon production, requiring the use of chemical pesticides with disease-resistant cultivars for control. However, the often rapid acquisition of fungicidal resistance by mildew pathogens makes this practice unsustainable. The identification of crop treatments that can enhance resistance to powdery mildew resistance is therefore important to reduce melon crop attrition. This study indicates that the application of Nano-Se can reduce the powdery mildew disease index by 21–45%. The Nano-Se treatment reduced reactive oxygen species (ROS) and malondialdehyde (MDA) accumulation, with increases in glutathione (GSH), proline and 1,1-Diphenyl-2-picrylhydrazyl radical (DPPH). Increases were also observed in the activities and transcriptional levels of the antioxidant enzymes superoxide dismutase (SOD), catalase (CAT), ascorbate peroxidase (APX) and peroxidase (POD). Assays with four different cultivars of melon with differing levels of mildew resistance demonstrated that relative to the control, the Nano-Se treatment resulted in larger responses to mildew infection, including increases in the levels of putrescine (PUT; 43–112%) and spermine (SPM; 36–118%), indoleacetic acid (IAA; 43–172%) and salicylic acid (SA; 24–73%), the activities of phenylalanine ammonium lyase (PAL), trans-cinnamate 4-hydroxylase (C4H) and 4-coumarate: Co A ligase (4CL) of the phenylpropanoid pathway (22–38%, 24–126% and 19–64%, respectively). Key genes in the polyamine and phenylpropanoid pathway were also upregulated. These results indicate that the foliar application of Nano-Se improved melon defenses against powdery mildew infection, with a significant reduction in mildew disease development.

**Graphical Abstract:**

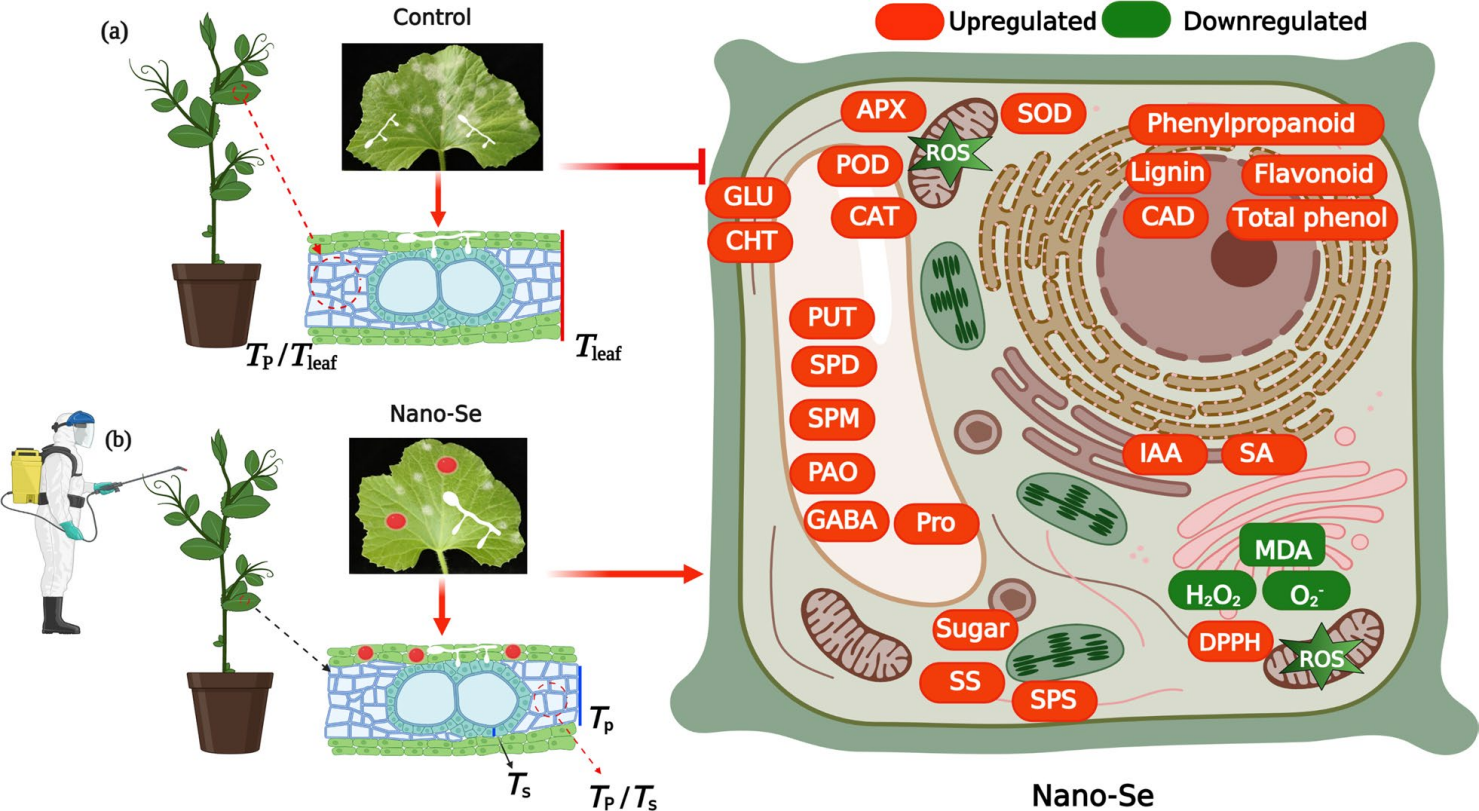

**Supplementary Information:**

The online version contains supplementary material available at 10.1186/s12951-023-02148-y.

## Highlights

1. Foliar spraying Nano-Se reduced the incidence of powdery mildew disease in melon.
2. Application of Nano-Se reduced alterations to leaf cell structure after powdery mildew infection.
3. Nano-Se foliar sprays increased reactive oxygen species (ROS) scavenging at the metabolic, enzyme activities and transcriptional levels.
4. Nano-Se promotes plant hormone signaling in leaves with increases in polyamine and phenylpropanoid metabolism.

**Supplementary Information:**

The online version contains supplementary material available at 10.1186/s12951-023-02148-y.

## Introduction

Melon (*Cucumis melo* L.) is an economically important crop [1] and China is the largest melon producer, accounting for 51% of the global production [2]. Melon has been cultivated in Xinjiang for more than 2000 years and the region is currently the largest melon producing region in China [3]. Powdery mildew, from either *Golovinomyces cichoracearum* or *Podosphaera xanthii*, can seriously affect melon yield and fruit quality throughout the year [4] in both field and greenhouse cultivations [5]. In China, the predominant mildew pathogen in melon has been reported to be *P. xanthii* [6]*.* The infection of melon by *P. xanthi* causes necrotic lesions in the leaves, which lowers the rate of net photosynthesis with reduced melon growth [7].

The pathogenesis of *P. xanthii* powdery mildew can be divided into three stages: the formation of adherent cells on the cell surface of host plants, the invasion of mycelia into host cells and the formation of fungal haustorium [8]. The haustoria are special infection structures formed in host epidermal cells for the absorbance of host cell nutrients, but also for the introduction of fungal effectors, which interact with and alters the plant biology to suppress defense responses and maintain the nutritional relationship with the host [9]. In the winter non-growing season, *P. xanthii* remains in the melon cultivation space in the form of mycelia, conidia and cleistothecia in the soil or as leaf bourn mycelia. As the temperature rises in the growing season, conidia infect new plant growth through air or water [10]. The use of chemical fungicides and the breeding of mildew resistant melon cultivars are currently used to control this disease [11]. However, the large-scale use of fungicides is environmentally damaging [12] and can rapidly lead to the selection of pathogen strains with fungicidal resistance. Furthermore, it is expected that the evolution of new strains of the pathogen will rapidly render the currently used mildew-resistant melon cultivars obsolete [13]. It is therefore necessary to seek alternative means to reinforce melon crop tolerance to powdery mildew disease.

Selenium (Se) compounds are known to have beneficial effects on plant resistance to pathogens [14], which may occur through a variety of mechanisms in different species, including the enhancement of reactive oxygen species (ROS) scavenging [15], defensive callose deposition [16] and the upregulation of antifungal pathways [17]. The application of Se to soil has also been reported to increase the relative abundance of beneficial microorganisms, with a decrease in pathogenic fungi [14]. Na_2_SeO_3_ was reported to directly impair fungal growth by damaging mycelial structure through effects on their chemical composition [18]. The dissolved organic matter in soil derived from Se-treated rape straw was shown to significantly inhibit the growth of *Sclerotinia sclerotiorum*, a soil borne pathogen of wide host range. This inhibition occurred with significant damage to its hyphae and reduction in its secretion of wall-degrading enzymes, implying its reduced capacity for plant infection [19]. The addition of low concentrations of selenite to traditional fungicides was suggested as a means to reduce the effective fungicidal dose in the control of *Fusarium* [20].

Nanoparticles are characterized by their small size, high surface area, surface charge, surface chemistry and solubility [21]. The use of nanotechnology in fertilizer production is considered promising for the improvement of food quality, food safety and crop growth [22]. Nano-Se can be prepared as a fertilizer supplement by reducing sodium selenite with ascorbic acid. These preparations have been shown to have protective effects against *S. graminicola* downy mildew disease [23] and wheat crown and root rot diseases induced by *Fusarium* species [24]. The foliar application of Nano-Se increased the resistance of citrus species to huanglong disease, with enhanced photosynthesis, antioxidant defense enzyme activities and non-enzymatic activities to maintain ROS balance [25]. However, how Se increases plant resistance to pathogens remains unclear and requires further study. The purpose of this study was to investigate (1) the effect of foliar Nano-Se treatment on melon resistance to powdery mildew disease; and (2) the physiological and biochemical bases of Nano-Se improvement of this resistance to powdery mildew infection. This study will provide insights into the mechanism of action of Nano-Se on crop resistance to fungal pathogens.

## Materials and methods

### Preparation and characterization of Nano-Se synthesized with ascorbic acid

Nano-Se was prepared as described previously [26]. Briefly, 10 mL of a 20 mM selenite solution and 4 mL of 1% ascorbic acid solution was added to 20 mL of 1% chitosan solution. The mixture was stirred at 500 rpm at 25 °C for 3 h until the solution became transparent red and Nano-Se colloids was prepared. Scanning electron microscopy (SEM) was conducted using a ZEISS Gemini SEM 300 (Oberkochen, Germany). SEM micrographs were obtained using the in-lens detector at 3.0 kV acceleration voltage. Transmission electron microscopy (TEM) utilized a JEOL JEM-F200 TEM microscope (Tokyo, Japan). The micrographs were obtained using the JED-2300 T energy spectrum at 200 kV acceleration voltage. Atomic force microscopy (AFM) image was acquired using Bruker Dimension Icon (Ettlingen, Germany) and processed with Gwyddion software v.2.43. The nanoparticle size and zeta potential distribution of Nano-Se were estimated via dynamic light scattering (DLS) using Malvern Zeta sizer Nano ZS90 (Worcestershire, UK). The DLS instrument was set at 25 °C with an adsorption coefficient of 0.01. The refractive index of the dispersive phase and dispersive environment was set to 1.59 and 1.33, respectively. X-Ray Diffraction (XRD) was conducted using a Bruker D8 Advance instrument (Ettlingen, Germany) and processed using XRD graphs were HighScore Plus software v.3.05. The spectrogram of Fourier-transform infrared spectroscopy (FT-IR) was acquired by Thermo Scientific Nicolet iS20 (Massachusetts, USA) between 4000 to 400 cm^−1^ at a resolution of 4 cm^−1^ and processed with OMNIC software v.8.2.

### Growth condition of melon and experimental design

Four melon cultivars of different resistance to powdery mildew were used in this research, including two susceptible cultivars, *Jia shi* (JSG) and *Zao zui xian* (ZZX), a moderately resistant cultivar, *Huang meng cui* (HMC) and the highly resistant cultivar, *Jun xiu* (JX) (the classification of their resistance was provided by the Melon Center of Xinjiang Agricultural science). Melon seedlings were cultivated in plastic flower pots (30 cm × 30 cm, two seedlings per pot) with organic soil (10–30 mm, Sphagnum peatmoss, Pindstrup Denmark) and watered regularly. After seedlings reached the two true leaf stage, seedlings were sprayed at weekly intervals with either water (control) or a Nano-Se solution (5.0 mg·L^−1^) for three weeks. Melon plants were treated with Nano-Se three times before pathogen inoculation. The control and 5 mg·L^−1^ Nano-Se treatment groups of the four melon cultivars each consisted of 16 pots. After the leaves were collected, the fresh and dry weight of the melon stems were weighed at 0 dpi (dried at 85 °C for 72 h).

### Pathogen inoculation, measurement of the incidence and severity of powdery mildew infection

Melon plants were treated with Nano-Se three times before pathogen inoculation. Conidia were collected in sterile water containing tween-20 (2 drops), and adjusted to 2 × 10^7^ /mL. For foliar inoculation, seedlings were sprayed with ca. 5 mL/ seedling of the conidial suspension, then covered in plastic film and placed in the dark for 24 h. The incidence of powdery mildew colonies per leaf and the powdery mildew index were measured at 4 and 9 days post inoculation (dpi). The disease index was investigated following the standards supplied by the Institute of Inspection and Testing for Pesticides, Ministry of Agriculture China [27]. The disease index was calculated according to the equation: Disease index (%) = [∑(*rn*_*r*_)/9*N*_*t*_] × 100, where r = rate value of leaf disease severity, n_r_ = number of diseased leaves with a rating of r, and N_t_ = total number of leaves tested (described in detail in [7]).

### Paraffin sectioning and toluidine blue staining of melon leaf material for microscopy

Leaves were collected at 1 and 10 dpi for tissue observation. The fresh tissue was placed in FAA fixative (Servicebio Biotechnology Co., Ltd, Wuhan, China) for 24 h, then processed for sectioning using an automatic tissue processor (Donatello, DIAPATH, Italy). Sections of 4 µm were prepared from paraffin wax-embedded tissues with a microtome (RM2016, Leica Instrument Co., Ltd, Shanghai, China). The sections were then rehydrated in a step series of alcohol (100–95–90–80%Ltd, Shanghai, China) then immersed in toluidine blue solution staining (Servicebio Biotechnology Co., Ltd, Wuhan, China) for 2 min, before rinsing under running water and clearing by three 5 min immersions in xylene (Sinopharm Group Chemical Reagent Co., Ltd, Shanghai, China). The sections were mounted with neutral balsam (Sinopharm Group Chemical Reagent Co., Ltd, Shanghai, China) and the images captured from an inverted light microscope (Eclipse E100, NIKON, Japan) equipped with a mounted DS-U3 digital camera (NIKON, Japan).

### ***Observation of hydrogen peroxide (H***_***2***_***O***_***2***_***) and superoxide radical (O***_***2***_^***−***^***) distribution in melon leaves                                                                                                                           ***

Leaves were collected at 2, 4, 8 and 10 dpi for observation of O_2_^−^ and at 4, 8 and 10 dpi for H_2_O_2_. Leaf O_2_^−^ and H_2_O_2_ were detected using nitro blue tetrazolium (NBT) and diaminobenzene (DAB) staining kits, respectively, as per the manufacturer’s instructions (Servicebio Biotechnology Co., Ltd, Wuhan, China), then placed in the dark for 2 h before image acquisition with a light microscope (Eclipse E200, Nikon, Japan).

### Scanning electron microscope (SEM) and transmission electron microscopy (TEM)

The leaves were collected at 9, 11 and 13 dpi after inoculation. The sample processing and image acquisition method for TEM were as described by Kang et al. [27]. For SEM, leaves were cut into 1 mm^2^ sections and fixed in 2.5% (v/v) glutaraldehyde for 5 days at 4 °C, before washing three times in phosphate buffer solution (PBS, pH 7.2–7.4) for 30 min. The tissues were fixed in 1% (v/v) OsO_4_ at room temperature for 2 h and washed three times for 30 min with PBS (pH 7.2–7.4). The tissues were dehydrated in a step gradient of ethanol solution (50, 70, 80, 90 and 100%) for 30 min at each step, freeze dried, tertiary butanol added, and placed at 4 °C. Tertiary butanol was frozen and dried in a freeze dryer (JFD-320, Japan), then the sample placed in the ion sputtering instrument (JFC-1600, Japan) for coating. A scanning electron microscope (JSM6390LV, Japan) was used to obtain the images.

### Determination of leaf contents of enzyme activities and metabolite levels associated with the oxidative stress, phenylpropanoid and carbohydrate metabolic pathways

The leaves were collected at 0, 10 dpi for the determination of various physiological and biochemical indexes. Melon leaves were ground in liquid nitrogen to a powder and suspended in extraction buffer consisting of PBS buffer at a ratio of 1 mL/initial leaf Fwt. The homogenates were clarified by centrifugation at 12,000*g* for 5 min at 4 °C. The leaf contents of enzyme activities, protein and metabolite levels (listed below) were determined using commercial assay kits as per their respective manufacturer’s instructions. Assay kits and their reference numbers from the Jiancheng Bioengineering Institute (Nanjing, China) were for superoxide dismutase (SOD; kit A001-3), catalase (CAT; A007-1-1), ascorbate peroxidase (APX; A123-1-1), peroxidase (POD; A084-3-1), chitinase (CHT; A139-1-1), phenylalanine ammonium lyase (PAL; A137-1-1), sucrose synthase (SS; A097-1-1), sucrose phosphate synthase (SPS; A098-1-1), H_2_O_2_ (A064-1-1), malondialdehyde (MDA; A003-1-1), proline (A107-1-1), radical scavenging/ antioxidant capacity (with 1,1-diphenyl-2-picrylhydrazyl radical; DPPH; A153-1-1), total phenols (A143-1-1), flavonoids (A142-1-1), sample protein concentrations (A045-4), total soluble sugar (A145-1-1), sucrose (A099-1-1), total amino acids (A026-1-1), glutamate (A074-1-1), glutamine synthetase (GS; A047-1-1) and lipoxygenase (LOX; H550-1). Assay kits from Solarbio Science & Technology (Solarbio Science & Technology Company Ltd, Beijing, China) were utilized to determine the enzyme activities of β-1,3-glucuronidase (GLU; BC0365), trans-cinnamate 4-hydroxylase (C4H; BC4080), 4-coumarate: Co A ligase (4CL; BC4220), the levels of reducing sugars (BC0235), lignin (BC4200), cinnamyl alcohol dehydrogenase (CAD; BC4170), and hydroxyproline (BC0255). Polyamine oxidase (PAO; ml092692) was measured with assay kits from Mlbio (Enzyme Linked Biological Reagent Technology Co., Ltd, Shanghai, China). GSH levels were determined with an assay kit from Elabscience (Elabscience Biotechnology Co., Ltd., Wuhan, China). The colorimetric or spectrophotometric results of assays were determined with either a UV–Vis spectrophotometer (Shunyu Hengping Instruments Co., Ltd, Shanghai, China) or a multiscan spectrum microplate spectrophotometer (Bio Tek Instruments Inc, USA).

### Ultra performance liquid chromatograph tandem mass spectrometry (UPLC-MS/MS) analysis of polyamines and γ-aminobutyric acid (GABA)

Frozen leaves were homogenized in 3 mL aqueous acetonitrile (8:2, v/v) and centrifuged at 4500 g for 10 min at 4 °C. The chromatographic conditions used were as follows: HILIC column (1.7 µm particle size; Waters, Milford, MA, USA); Mobile phase: (A) 5 mmol/L ammonium acetate acetonitrile solution and (B) 0.1% formic acid; gradient elution: 0–1.0 min, 80% A; 1.0–1.5 min, 80%–40% A; 1.5–6.0 min, 40% A; 6.0–6.5 min, 40%–80% A; 6.5–9.5 min, 80% A. Injection volume: 2 µL; flow rate: 0.3 mL/min; column temperature 30 °C. In positive ion mode, the ion source temperature was 150 °C and the dissolvent temperature was 350 °C. The flow rate of N_2_ and N_2_ cone hole gas was set to 650 L/h and 250 L/h, respectively. The acquisition parameters used for PUT, SPM, SPD and GABA are shown in Additional file 2: Table S1.

### UPLC-MS/MS analysis of the plant hormones, jasmonic acid (JA), salicylic acid (SA) and indoleacetic acid (IAA)

Frozen leaves were homogenized in 1 mL of methanol/water/formic acid (15:4:1, v/v/v) and centrifuge at 16,000 ×*g* for 5 min at 4 °C. The chromatographic conditions for the separation of the three plant hormones were as described in Kang et al. [28]. UPLC*-*MS/MS (Xevo TQ-S micro, Waters, Milford, MA, USA) spectra of JA, salicylic acid SA and IAA were acquired in the negative ion mode, with an ion source temperature of 150 °C and dissolvent temperature of 350 °C. The flow rate of N_2_ and N_2_ cone hole gas were set to 650 L/h and 250 L/h, respectively. The acquisition parameters used for JA, SA and IAA are shown in Additional file 2: Table S1.

### Quantitative real-time polymerase chain reaction (qRT-PCR) analysis

Total RNA was extracted from melon seedling leaves using an RNA simple total RNA kit (Tiangen Biotech Company Co., Ltd., Beijing, China). First strand cDNA, synthesis utilized the kit and platform of Transgene Biotech as per the manufacturer’s instructions (Transgene Biotech Company Ltd., Beijing, China). The primer sequences for target and reference (actin; XM_008442791.2) genes are shown in Additional file 2: Table S2. Relative mRNA levels were calculated using the 2^–ΔΔCt^ method [29].

### RNA-seq analysis

All samples were frozen in liquid nitrogen immediately after harvesting and stored at − 80 °C until processing. Each sample had three biological replicates. Total RNA was isolated using the RNA simple total RNA kit (Tiangen Biotech Company Co., Ltd., Beijing, China) according to the manufacturer’s instructions. The quality of the extracted RNA was inspected by UV/VIS spectrometry (NanoPhotometer® spectrophotometer; IMPLEN, CA, USA) and 1% agarose gel electrophoresis to determine the RNA degradation and contamination. RNA samples were sent to Metware Co., Ltd. (Wuhan, China) for transcriptome sequencing on an Illumina HiSeq 4000 platform. Raw reads were first analyzed by Fastq (version 0.19.3) for data quality control. Reads containing adapters, sequences exceeded 10% N content or of low-quality (Q ≤ 20) were removed. All subsequent analyses were based on the high-quality clean data. Reads were mapped and annotated to the reference Melon Genome (DHL92) v3.6.1 assembly transcript (http://cucurbitgenomics.org/organism/18), using HISAT (version 2.1.0). All 24 libraries (JSG-CK, HMC-CK, JSG and HMC with Nano-Se at 0 dpi and JSG-CK, HMC-CK, JSG and HMC with Nano-Se at 10 dpi, three replicates for each treatment) were combined into one pool. Enrichment analyses were performed based on the hypergeometric test.

### Statistical analysis

SPSS (version 26 SPSS Inc., Chicago, IL) was used for one-way analysis of variance (ANOVA). Student-Neuman-Keuls tests were used for multiple comparisons for significant differences (*p* < 0.05). Graph Pad Prism software (version 8.02; San Diego, CA) was used for the calculation of other statistics and for data plots. For transcriptome analysis, DESeq2 (version 1.22.1) was used to calculate the fragments per kilobase of transcript per million mapped reads (FPKM) and to identify differentially expressed genes (DEGs). The P value was corrected using the Benjamini & Hochberg method. DEGs were defined as those displaying a log_2_ fold change > 1 and a false discovery rate (FDR) < 0.05. The DEGs were then annotated with their Gene Ontology (GO) and Kyoto Encyclopedia of Genes and Genomes (KEGG) terms. All the data analyses were performed in the R environment (https://www.r-project.org/).

## Results and discussion

### Characterization of the synthesized Nano-Se

The synthesis of Nano-Se in this study utilized ascorbic acid and chitosan to prevent the aggregation of nanoparticles and improve the bioavailability of Nano-Se [30]. The synthetic process was taken as complete after solution became red (Additional file 1: Fig. S1A). In order to study the average particle size and shape of the synthesized Nano-Se, SEM and TEM observations were conducted. Dynamic Light Scattering (DLS) analysis was also used to define the particle size distribution and polydispersity index (PDI) [31]. The surface characteristics and morphology of the synthesized Nano-Se were studied by SEM and TEM. Sodium selenite was dispersed by chitosan and ascorbic acid, and the surface of Nano-Se was uniform and clear. SEM (Additional file 1: Fig. S1B) and TEM (Additional file 1: Fig. S1G, J) images monodisperse spherical Nano-Se with a size between 50 and 200 nm. The morphology was further validated by AFM (Additional file 1: Fig. S1K). DLS technology defined the typical particle size distribution, which was estimated to be 72.4 nm (Additional file 1: Fig. S1M). The polydispersity index (PDI) calculated from DLS data, where PDI values < 0.05 are fit best to the monodisperse model, whereas PDI values > 0.7 are considered to indicate a polydispersity diffusion of particles [32]. In our study, the synthesized Nano-Se has a PDI value of 0.085, indicative of a medium dispersity. Energy dispersive X-ray (EDX) research is a method used for elemental analysis [33]. The basic structure and purity of the synthesized Nano-Se were determined by EDX, as shown in Additional file 1: Fig. S1D–J. The specific absorption peak of Nano-Se (1.74 keV) corresponds to Selenium, with no other element peak detected in the spectrum (Additional file 1: Fig. S1E). EDX mapping confirmed the composition of the Nano-Se, indicating the presence of C, O and Se (Additional file 1: Fig. S1H–J). The X-ray diffraction (XRD) spectra were consistent with the Se phase (Additional file 1: Fig. S1N). Additional file 1: Fig. S1O depicts the FTIR spectrum of the synthesized Nano-Se, which shows an expanded band width of 3266 cm^−1^ (≡C–H stretching band) indicating the existence of the alkyne. The medium-strong spike evident at 1637 cm^−1^ is caused by carbonyl C = O, confirming the presence of an amide group.

### Effects of Nano-Se on the incidence of powdery mildew disease in melon leaves

**Fig. 1:**
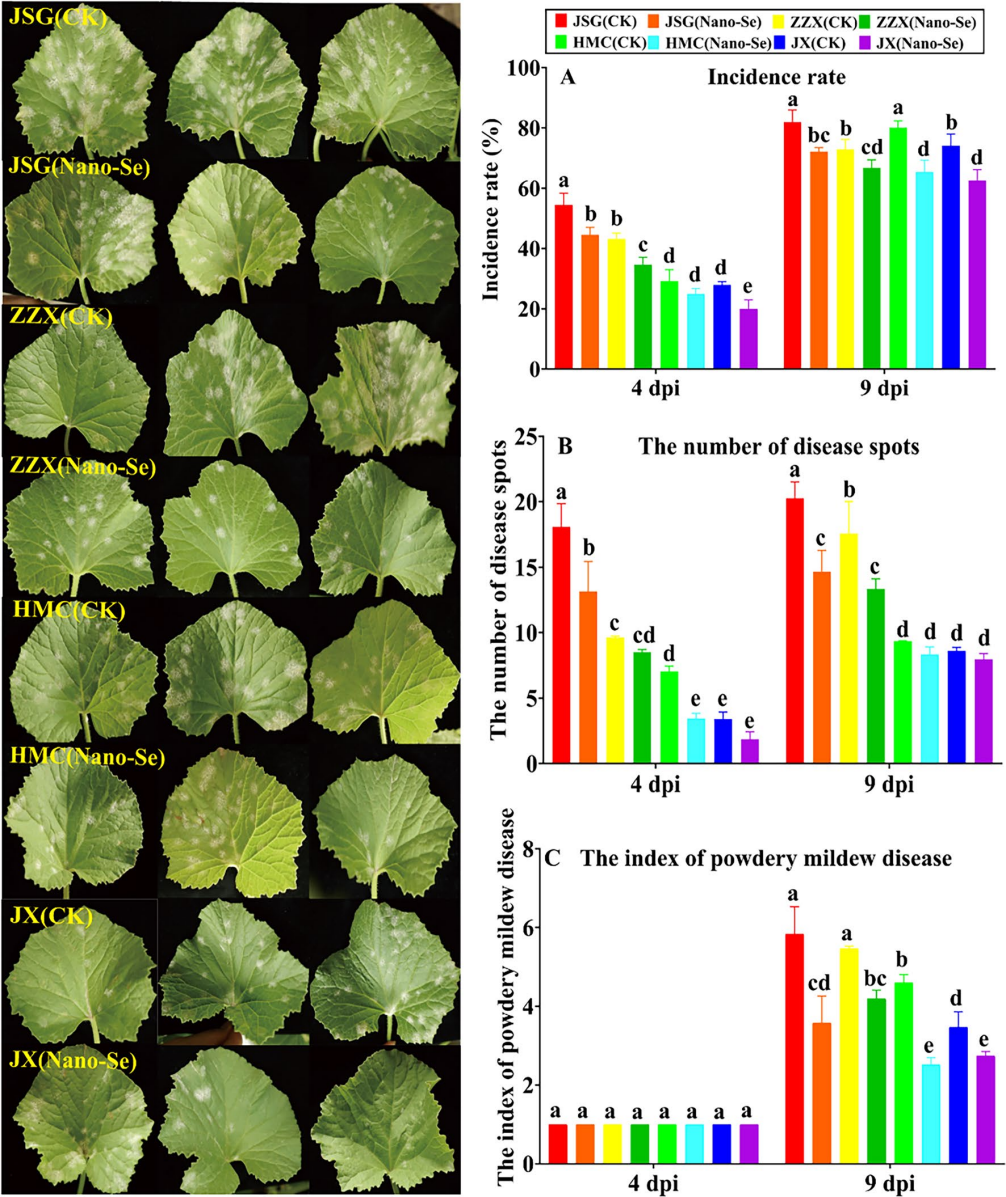
Effect of Nano-Se on the incidence of powdery mildew in leaves of melon cultivars of differing resistance at different infection stages. JSG, ZZX, HMC and JX represent the four melon cultivars with different resistances to powdery mildew, which are *Jia shi*, *Zao zui xian* (susceptible) and *Huang meng cui*, *Jun xiu* (resistant), respectively. CK and Nano-Se represent the control leaves and those sprayed with 5.0 mg⋅L^−1^ Nano-Se, respectively. dpi: days post inoculation. The different lowercase letters above the bars indicate a significant difference (*p*＜ 0.05) between the treatments and the error bars represent standard deviations (n = 4)

The effects of the Nano-Se treatment on the incidence of powdery mildew in melon cultivars of differing resistances is shown in Fig. 1. At 4 dpi, the Nano-Se treatment significantly reduced the incidence of powdery mildew in JSG, ZZX and JX plants by 18, 20 and 28%, respectively. At 9 dpi, the incidence rate of powdery mildew in JSG, ZZX, HMC and JX plants were significantly reduced by 12, 8, 18 and 16%, respectively (Fig. 1A). Relative to their controls, the Nano-Se treatment significantly reduced the number of disease spots in JSG and HMC plants by 27 and 51%, respectively and at 9 dpi by 28 and 24% in JSG and ZZX plants, respectively (Fig. 1B). No significant effect of the Nano-Se treatment on the index of powdery mildew disease could be detected at 4 dpi, whereas at 9 dpi, the index was significantly reduced in all four cultivars by 21–45% (Fig. 1C). Nano-Se was reported to an effective and economic alternative to control fungal plant pathogens of faba bean [34].

Nano-Se treatments of soil or plants can enhance plant resistance to fungal pathogens [35–37]. However, how Nano-Se achieves this protective effect remains unclear. In this study, Nano-Se was shown to reduce the disease index in melon infected with a main agent of powdery mildew, *P. xanthii* by 21–45% (Fig. 1) and that this occurred with increases in active oxygen species scavenging, polyamine synthesis, phenylpropanoid metabolism and changes in plant hormone levels. The effects of the foliar application of Nano-Se on the melon stem weights in the four melon cultivars are shown in Additional file 2: Table S3. Compared with the control group, the Nano-Se treatment significantly increased the fresh weight of stems in JSG, ZZX and JX plants at 0 dpi by 62, 76 and 24%, respectively, and increased dry weight of stems in JSG, ZZX and HMC plants by 79, 23 and 93%, respectively.

### Effect of the Nano-Se treatment on structural alterations in melon leaves infected with *P. xanthii*

#### The alterations in melon leaf anatomical organization after powdery infection are reduced in Nano-Se treated plants

**Fig. 2:**
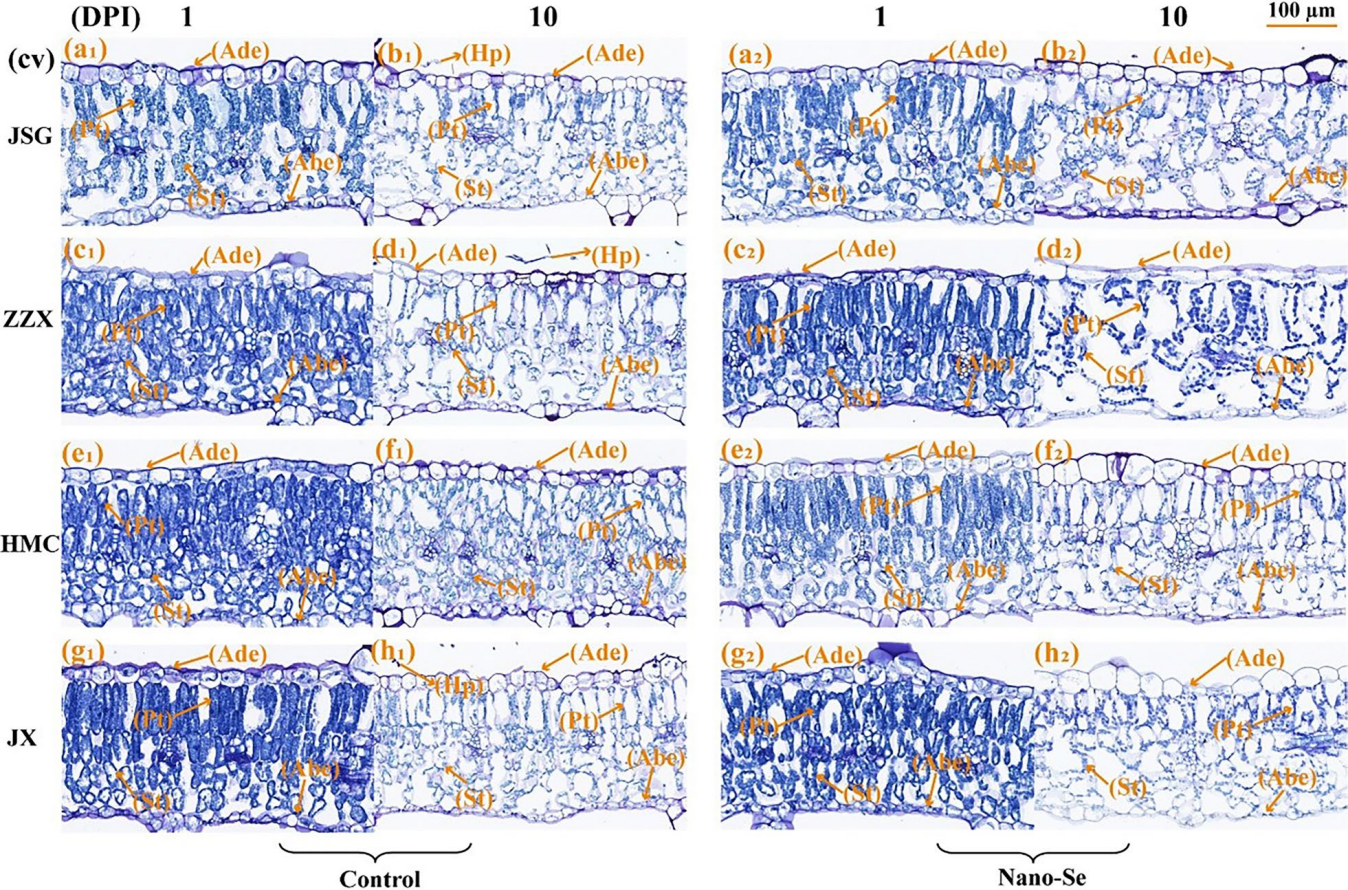
Anatomical structural characteristics of leaf mesophyll cells infected with powdery mildew at 1 and 10 dpi in susceptible and resistance cultivars with or without Nano-Se treatment. The panels (a_1_, a_2_, b_1_, b_2_), (c_1_, c_2_, d_1_, d_2_) and (e_1_, e_2_, f_1_, f_2_), (g_1_, g_2_, h_1_, h_2_) represent the paraffin section images of powdery mildew susceptible (JSG, ZZX) and resistant cultivars (HMC, JX) at 1, 10 dpi in the control and Nano-Se treatment group, respectively. Key: Ade: adaxial epidermis; Abe: abaxial epidermis; Pt: Palisade tissue; Sp: Spongy tissue; Hp: Hyphae

Leaf structure is affected by and can adapt to changing environments [38, 39]. As shown in Fig. 2, the anatomical structure of melon leaves largely consists of the (top to bottom) adaxial epidermis (Ade), palisade tissue (Pt), spongy tissues (St) and the abaxial epidermis (Abe). The Ade and Abe are both cellular monolayers. The Ade cells were flat and nearly rectangular, whereas those of the Abe were small, irregular in shape and tightly packed. The tissue compactness after the Nano-Se treatment was higher than that of the control group (Additional file 2: Table S4), and a large amount of mycelium was attached to the adaxial epidermis of the control leaves of the susceptible cultivar at 10 dpi. The tissue sections of inoculated melon leaves (Fig. 2b1, d1, h1) clearly showed a large number of hyphae (Hp) attached to the upper surface of leaf epidermal cells at 10 dpi, while no invading hyphae were observed in the Pt or St. At 1 dpi, the tissue structure of melon leaves (Fig. 2a1–h1) showed an orderly arrangement to the Pt cells and a relatively compact St. However, at 10 dpi (Fig. 2a2–h2), the Pt and St were loosely arranged.

The anatomical characteristics of infected melon leaves at 1 dpi were shown in Additional file 2: Table S4. Leaf thickness, stomatal density and spongy tissue thickness significantly affected the resistance of loquat leaf spot disease [40]. The leaf thickness of all four cultivars were significantly increased in the Nano-Se treated plants by 11–34%. The thickness of the Ade of HMC and JX leaves was also significantly increased after Nano-Se treatment by 33 and 31%, respectively. The palisade tissue thickness of ZZX and JX Nano-Se treated leaves significantly increased by 17 and 21%, respectively. The anatomical characteristics at 10 dpi melon leaves are shown in Additional file 2: Table S4. Relative to their controls, the Nano-Se treatment resulted in significantly increased leaf thickness in JSG, HMC and JX cultivars by 12, 17 and 42%, respectively. The Ade thickness of JSG after Nano-Se treatment significantly increased by 41%. In the Nano-Se group, the thickness of the Abe and Pt layers in HMC and JX leaves showed significant increases of 53, 17, 37 and 73%, respectively. In ZZX, HMC and JX, the Nano-Se treatment significantly increased the Pt to St ratio by 63, 33 and 20%, and the tissue compactness by 50, 17 and 24%, respectively.

#### Effects of Nano-Se foliar treatment on fungal development and melon leaf cell ultrastructural changes after infection with *P. xanthii*

Scanning electron microscopy (SEM) observations of melon leaves at different stages of powdery mildew infection are shown in Additional file 1: Fig. S2. To highlight the ultrastructural effects of *P. xanthii* and the amelioration of such by Nano-Se, only the susceptible cultivars were studied. As the infection progresses the mycelial network becomes denser, with greater hyphal leaf penetration and the production of many conidiophores. When the mycelium invades into the leaves, it produced haustoria, which may have an impact on leaves. Compared with the control plants, the mycelial density on the leaf surface of Nano-Se treated plants was reduced. In the present research, Nano-Se was shown to help maintain the structure of the palisade and spongy tissue layers and to reduce the number of mycelia detected by SEM in these plants (Additional file 1: Fig. S2a2–f2).

**Fig. 3:**
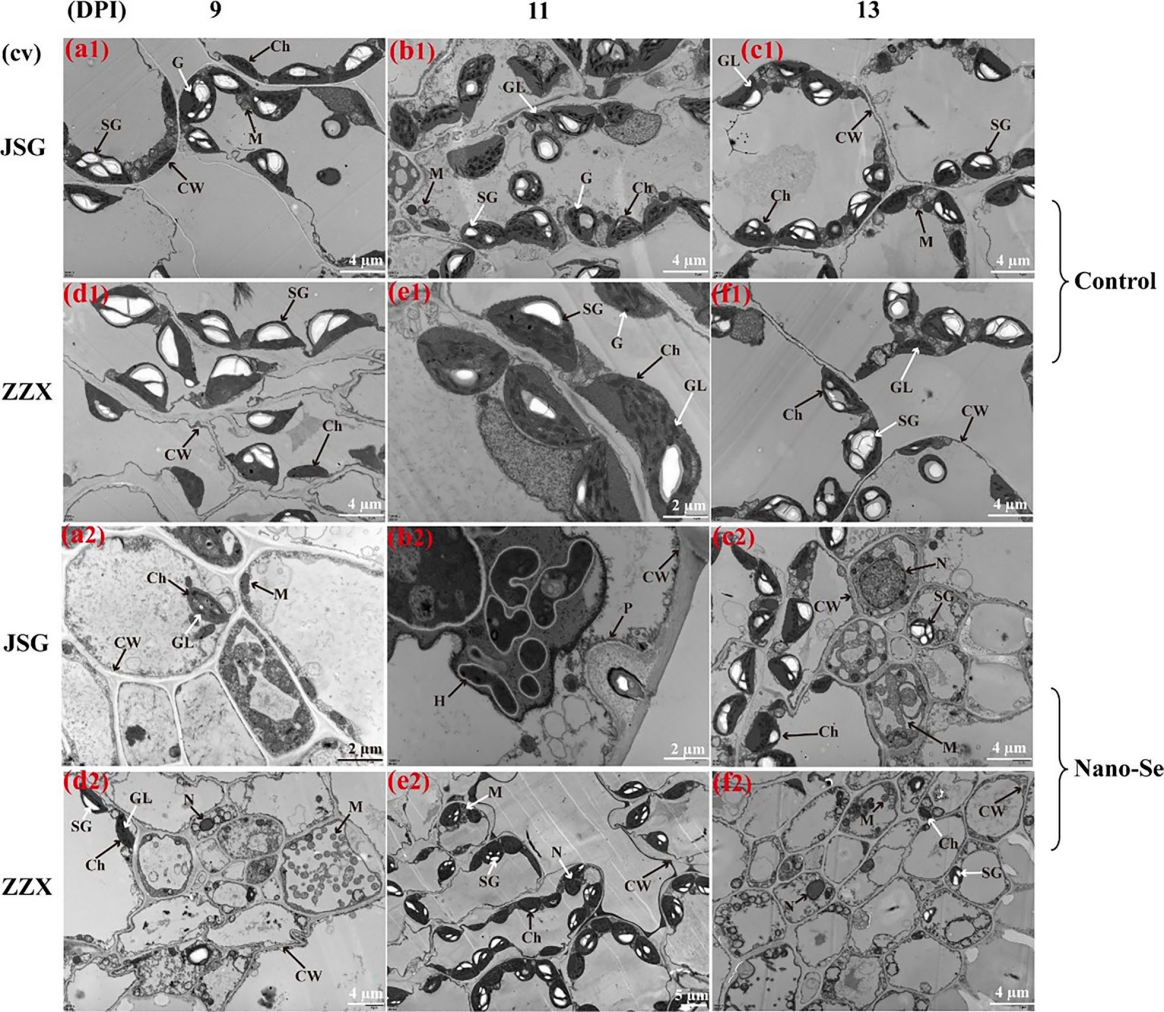
Transmission electron microscopic (TEM) of control and Nano-Se treated melon leaves at different infection stages of powdery mildew infection. (a), (b), (c), (d), (e) and (f) represent the cultivars JSG and ZZX at 9. 11 and 13 dpi, respectively. Panels 1 and 2 represent the control and treated leaves (5.0 mg L^-1^), respectively

The cell ultrastructural characteristics of melon leaf cells were studied with transmission electron microscope (TEM) at different stages of powdery mildew infection (Fig. 3). As the infection progressed, the palisade chloroplasts swelled, became more spherical and clumped together. With the increase in chloroplast deformation, the starch granules changed from long strips to oval or triangular, the osmiophilic granules in cells increased, and the grana lamellar structure changed significantly (panels b1, c1–2, f1). The cell wall serves as a primary defense against fungal invasion and is rapidly and locally modified in response to fungal infection, including the formation of encapsulating papillae [41]. In the early stage of fungal contact and cell penetration, plants cells form a papilla between the cell wall and the plasma membrane to impede fungal penetration. An example of this is given in panel b_2_. However, no papillae were found in JSG leaves of the control group, which was consistent with the SEM results. The intrusion plugs of powdery mildew penetrate the papillae to form haustoria. The epidermal cells of JSG leaves treated by Nano-Se were filled by multiple haustoria at 11 dpi. The papilla structure was observed in Nano-Se treated plants by TEM (Fig. 3b2), which indicated that the Nano-Se pretreatment enhanced the resistance of melon plants.

### Effects of Nano-Se on melon leaf chitinase (CHT) and β-1, 3-glucanase (GLU) after infection with *P. xanthii*

**Fig. 4:**
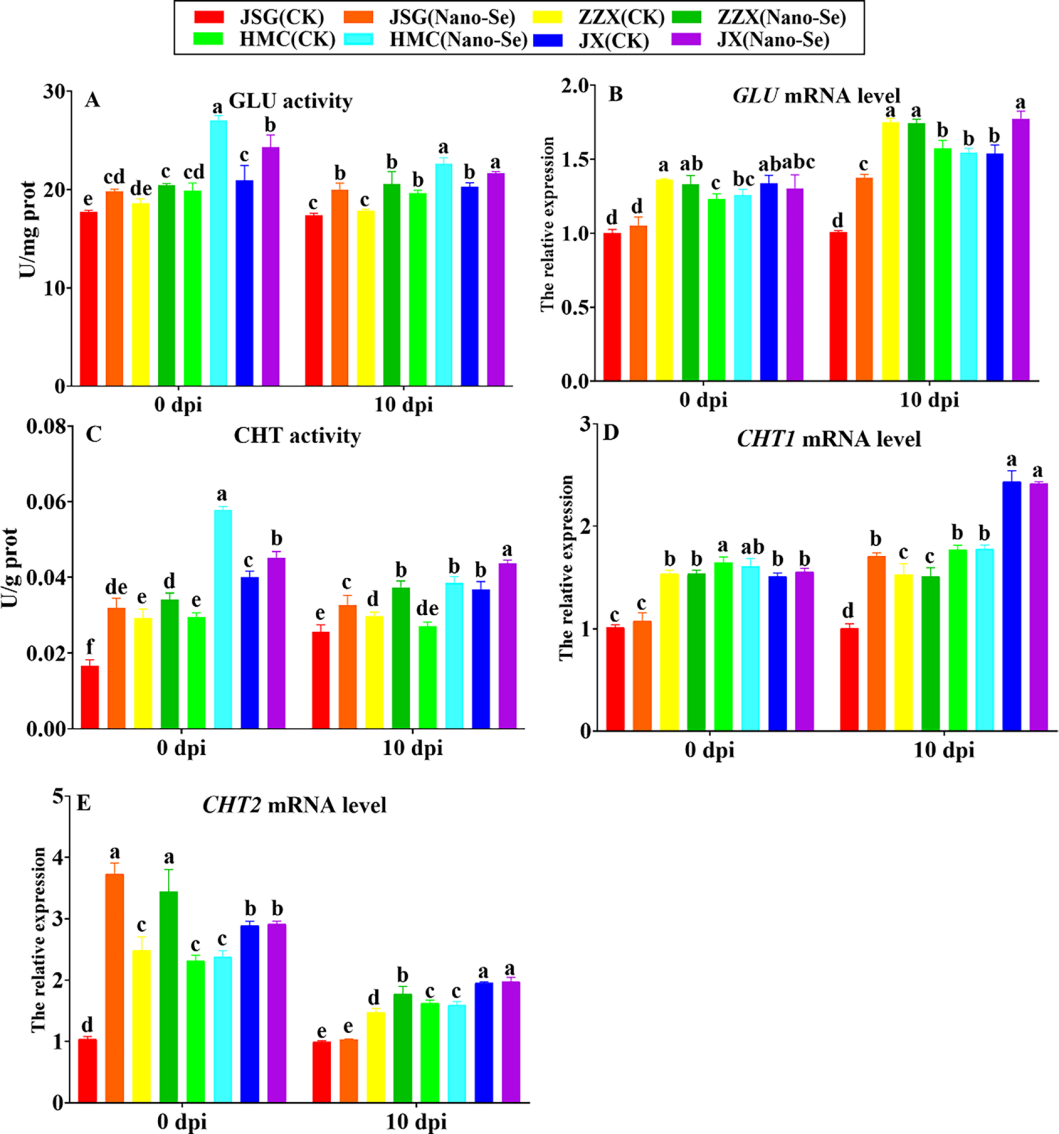
Effects of Nano-Se on leaf defense enzyme activity and mRNA levels at 0 and 10 dpi. The figure layout, cultivars and conditions utilized are as in Fig. 1

Pathogenesis-related (PR) proteins are important components of plant defense [42]. Members of the GLU and CHI PR families catalyze the degradation of fungal cell walls [43]. The influence of Nano-Se on melon leaf CHT and GLU at the levels of enzyme and transcriptional activity is shown in Fig. 4. Prior to infection (0 dpi), Nano-Se treatment resulted in significantly higher GLU activities in all four cultivars (10–36%). At 10 dpi, Nano-Se treatment also significantly increased GLU activity (7–15%) in four cultivars. Before infection (0 dpi), the levels of *GLU* mRNA were largely unaffected by Nano-Se treatment in all four cultivars. At 10 dpi, *GLU* mRNA levels in ZZX and HMC remained largely unaffected, while *GLU* mRNA levels in JSG and JX were significantly increased by 15–36%. GLU activity was shown to induce the systemic response in plants by releasing polymers from the fungal cell wall [44].

Chitin is one of the main components of fungal cell walls consisting of a homopolymer of N-acetyl-D-glucosamine linked through β (1–4) bonds and can induce the plant defensive response [45]. Nano-Se treatment significantly increased CHT activities at 0 dpi (12–95%) and at 10 dpi, (19–42%) in the four cultivars. At 10 dpi, Nano-Se significantly upregulated the expressions of *CHT1* in JSG and *CHT2* in ZZX by 70% (D) and 20% (E), respectively. Conversely, Nano-Se treatment group significantly increased *CHT2* expression in JSG and ZZX at 0 dpi by 259 and 38%, respectively. In this study, Nano-Se was shown to increase the activities and mRNA levels of GLU and CHI (Fig. 4), indicating they could play a role in the Nano-Se mediated enhanced resistance to *P. xanthii*. Sugar signaling is closely related with cell proliferation, plant growth and development and interacts with many other signal pathways [46].

### Effects of Nano-Se treatment on LOX and plant hormones in melon leaves infected with *P. xanthii*

The effects of Nano-Se treatment groups on LOX activity and mRNA levels of melon leaves infected with powdery mildew were not significant (Additional file 1: Fig. S3). The effects of Nano-Se on plant hormone levels in melon leaves inoculated with powdery mildew pathogens are shown in Additional file 1: Fig. S4. Infection was seen to decrease the IAA levels in control plants without the Nano-Se treatment. Conversely, Nano-Se treatment significantly increased the IAA content in the four cultivars at both 0 dpi (28–124%) and 10 dpi cultivars (43–172%). IAA treatment increased the resistance of kiwifruit to *Botrytis cinerea*, with the promotion of high activities of pathogen resistance-related defense enzymes [47].

The JA and SA signaling pathways have been reported to play key and integrated roles in the poplar response to fungal infection [48]. The leaf SA content was also increased in response to infection in the control group without the Nano-Se treatment. However, relative to the control levels, the SA levels in Nano-Se treated plants were significantly increased the SA content in the four cultivars at both 0 dpi (61–122%) and 10 dpi (24–73%). Chitosan enhanced the resistance of apple to Glomerella leaf spot through the SA signal pathways [49], which was consistent with this study (Additional file 1: Fig. S4). The synthesis and signal transduction of JA pathway were inhibited during the infection of *Botrytis cinerea* [50]. In contrast, although infection with *P. xanthii* induced higher JA levels, the Nano-Se treatment had no further significant effect. In this study, there was no significant difference in gene expression involved in JA synthesis, including *LOX1*, *LOX2*, *LOX8*, *LOX9* and *LOX10* (Additional file 1: Fig. S3). The upregulation of gene expression in plant hormone signal pathways, including those of SA and JA, has been reported to be involved in fruit defense against molds [51]. SA was shown to activate catechin and proanthocyanidins biosynthesis in poplar trees against rust infection [52].

### Effects of Nano-Se on antioxidant capacity of melon leaves infected with *P. xanthii*

**Fig. 5:**
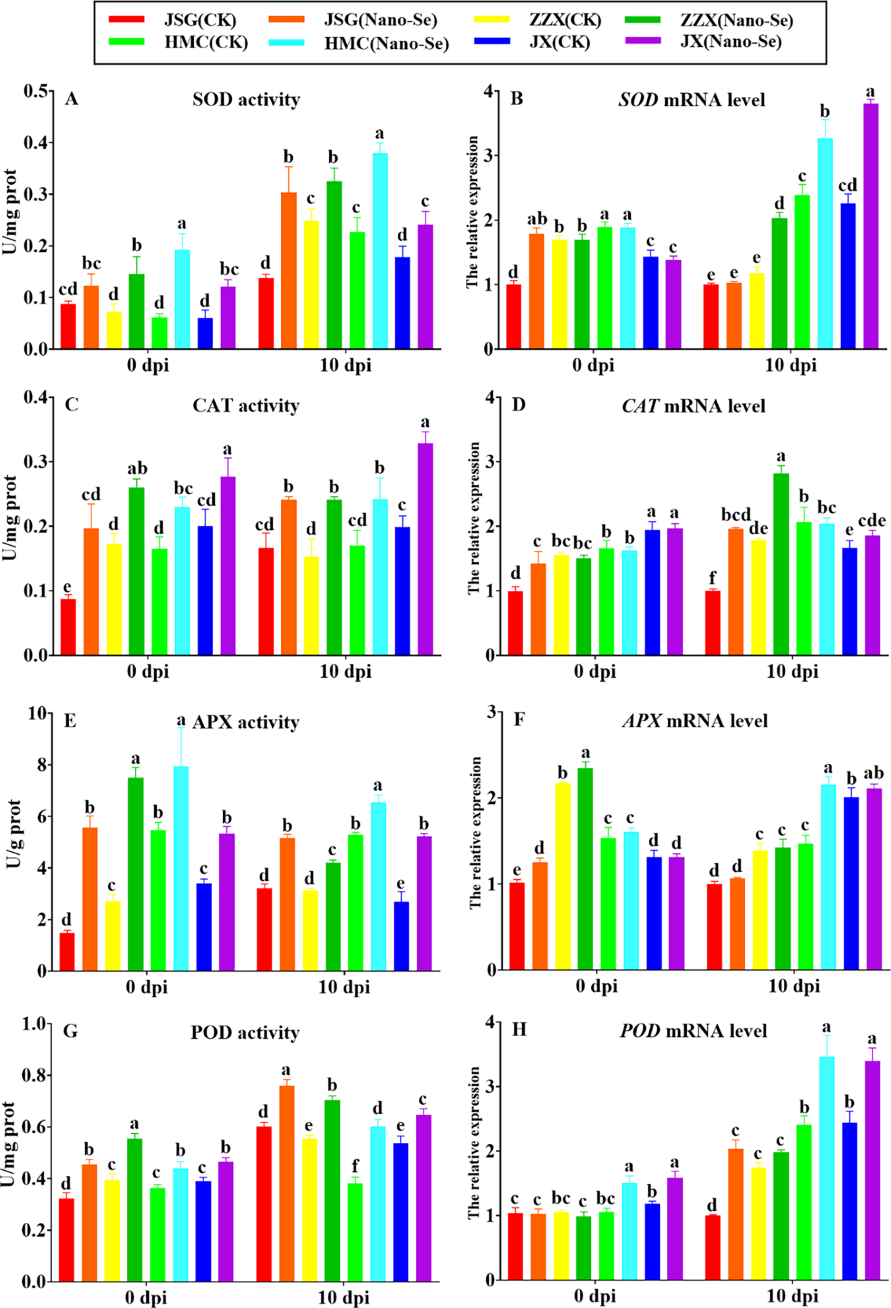
Effect of Nano-Se on leaf antioxidant activities at 0 and 10 dpi. The figure layout, cultivars and conditions utilized are as in Fig. 1

The effects of Nano-Se on the leaf antioxidant enzyme activities before (0 dpi) and after (10 dpi) *P. xanthii* infection are shown in Fig. 5. Nano-Se treatment resulted in a significant increase in SOD activity in all four cultivars. Both prior to (41–215%) and following infection (31–120%; A). Before infection (0 dpi), *SOD* mRNA levels were largely unaffected by Nano-Se in ZZX, HMC, and JX, but significantly increased in JSG by 44%. In contrast, at 10 dpi, Nano-Se treatment significantly increased *SOD* mRNA levels in ZZX, HMC and JX (37–72%; B). Quiterio-Gutierrez et al. [53] showed that the combined application of Nano-Se and Nano-Cu reduced the incidence of *Alternaria solani* in tomato plants, with the induction of higher leaf activities of SOD, APX, glutathione peroxidase (GPX) and PAL. Nano-Se treatment increased CAT activity both before (38–126%) and after infection (42–65%; C) in the four cultivars. Before infection (0 dpi), the *CAT* mRNA levels were largely unaffected by Nano-Se in ZZX, HMC and JX, but significantly increased by 30% in JSG. At 10 dpi, the Nano-Se treatment resulted in significant increases (58–96%) in *CAT* mRNA levels in JSG and ZZX, but not in HMC or JX (D). Nano-Se treatment increased APX activity (45–279%) in the four cultivars both before (45–279%) and after infection (24–94%; E).

Before infection (0 dpi), Nano-Se treatment resulted in increased *APX* mRNA levels were increased by Nano-Se before infection in JSG and ZZX (8–23%). In contrast, at 10 dpi, Nano-Se treatment significantly increased *APX* mRNA levels only in HMC (32%; F). POD activity was increased by Nano-Se in all four cultivars prior to (19–41%) and following infection (21–58%; G). Increases in enzymes involved in ROS scavenging, including POD, has been linked to the improved disease resistance of loquat fruit to *C*. *gloeosporioides* [54]. However, before infection (0 dpi), Nano-Se treatment resulted in increased *POD* mRNA levels were only increased prior to infection in HMC and JX (34–43%) and after infection in JSG, HMC and JX (28–51%; H). Following infection, the antioxidant capacity of disease-resistant cultivars was stronger than in disease-susceptible cultivars in the control group without Nano-Se application. The antioxidant capacity of melon seedlings treated with Nano-Se increased both before and after infection with *P. xanthii* powdery mildew, but to different degrees.

**Fig. 6:**
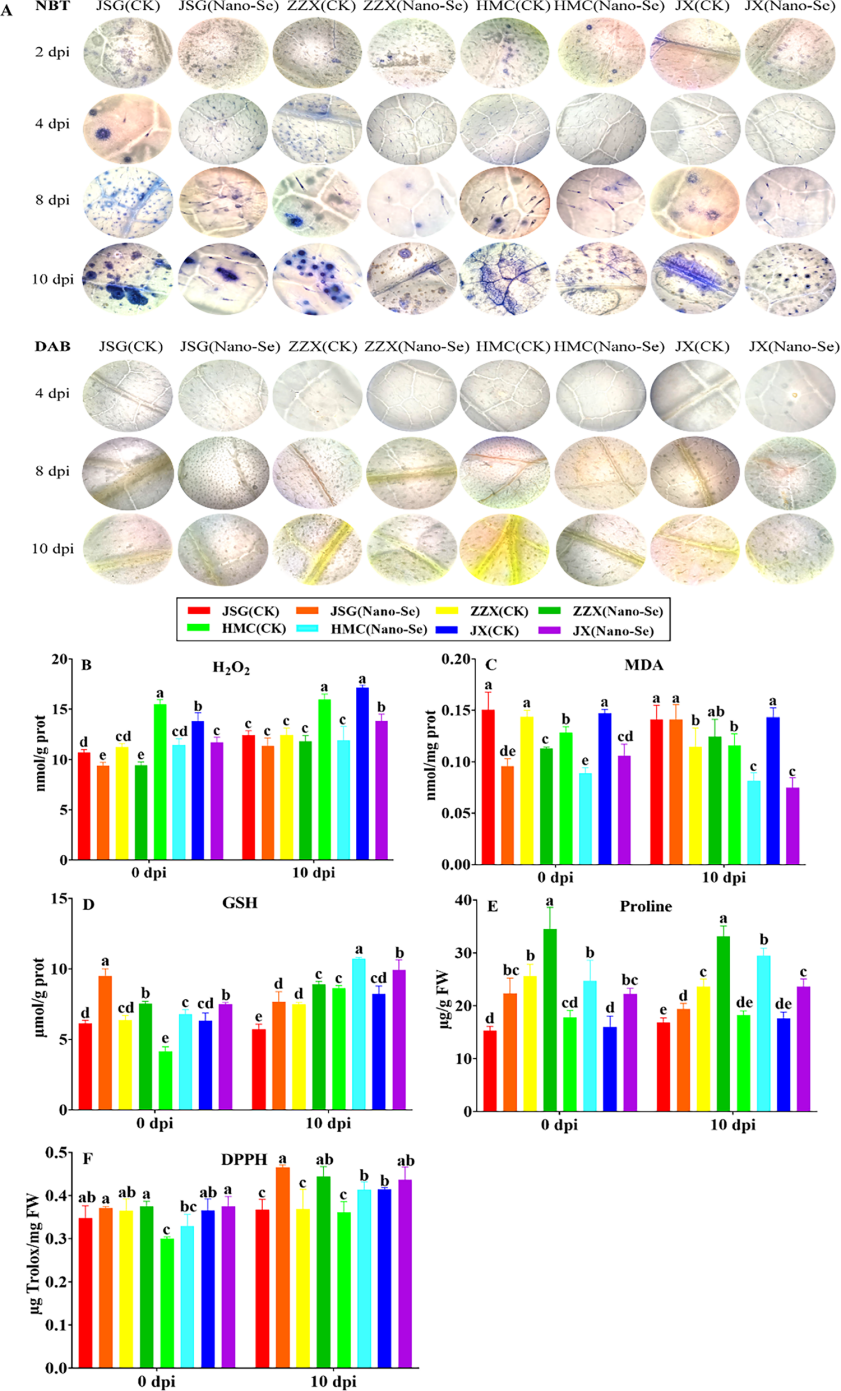
Effect of Nano-Se on leaf ROS accumulation and lipid peroxidation at 0 and 10 dpi. The figure layout, cultivars and conditions utilized are as in Fig. 1

The immune response of plants to pathogens can involve callose deposition, the accumulation of ROS and cell death [55]. The effect of Nano-Se on ROS accumulation in leaves at different ages of powdery mildew infection are shown in Fig. 6. Tissue observations of H_2_O_2_ and O_2_^−^ at 2, 4, 8 and 10 dpi (Fig. 6A) showed that both ROS were induced to higher levels after infection, indicating a higher level of oxidative stress, but that increases in O_2_^−^ were detectable after 2 dpi, whereas increases in H_2_O_2_ were seen only after 8 dpi. Reactive oxygen species (ROS) rapidly accumulate in plants infected by microbial pathogens, and are important components of the plant defense response [56]. Notably, the accumulation of both ROS was reduced by the Nano-Se treatment in all cultivars. To further examine the effect of Nano-Se on the antioxidant capacity, radical scavenging and lipid peroxidation activities in melon leaves during powdery mildew infection, the levels of H_2_O_2_, MDA, GSH, proline and free radical scavenging/antioxidant activity (using DPPH) were determined (Fig. 6B–F). Fungal ROS metabolism is central to the infection process, and crucial in plant colonization [57]. Nano-Se was seen to increase leaf activities of SOD, CAT, APX and POD as well as their gene expression (Fig. 5), which occurred with a reduction in H_2_O_2_, O_2_^−^ and MDA and an increase in GSH, proline and radical scavenging capacity (Fig. 6).

The regulation of ROS levels in pathogen-challenged plants plays a decisive role between susceptible and resistant interactions [58]. In confirmation of histological observation (Fig. 6A), Nano-Se treatment significantly reduced H_2_O_2_ accumulation in leaves at 0 and 10 dpi in all cultivars by 12–26% and 9–25%, respectively (B). Bai et al. [59] showed that H_2_O_2_ accumulation increased significantly with the decrease of total antioxidant capacity. Nano-Se significantly increased the levels of GSH at both 0 and 10 dpi in the all four cultivars by 18–63% and 19–34%, respectively (D). Nano-Se also promoted a significant increase in proline levels at both 0 and 10 dpi in all cultivars by 39–46% and 16–61%, respectively (E). Based on the DPPH assay, Nano-Se significantly increased the antioxidant activity at both 0 and 10 dpi in all cultivars by 3–10% and 5–27%, respectively (F). Nano-Se reduced the leaf MDA content by 21–36% at 0 dpi, and by 30 and 48% at 10 dpi in cultivars HMC and JX, respectively (C). Wang et al. [60] reported that Na_2_SeO_3_ reduced H_2_O_2_ and MDA levels in alfalfa and Cunha et al. [61] demonstrated that Na_2_SeO_4_ enhanced enzymatic and non-enzymatic antioxidant metabolism in peanut plants.

### Effects of Nano-Se on the primary metabolism of *P. xanthii* infected melon leaves

#### Sugar content

Higher sugar contents have been reported to induce higher pathogen resistance in plants by promoting cell wall lignification, flavonoids synthesis and the levels of PR proteins [62]. Prior to the onset of infection (0 dpi), Nano-Se treated plants of all cultivars showed significant increases in leaf soluble sugar, reducing sugar and sucrose contents by 16–36%, and 17–40% and 22–65%, respectively (Additional file 1: Fig. S5A, B, C). The increased levels of sucrose were accompanied by increases in both sucrose phosphate synthase (52–206%) and sucrose synthase (24–91%) activities (D, E). At 10 dpi, the contents of soluble sugar, reducing sugar, sucrose and sucrose synthase activity were all decreased, but not significantly different between the control treatment without Nano-Se application and the Nano-Se treatment. In our study, at the early stage of powdery mildew infection, Nano-Se was seen to promote increases in soluble and reducing sugars, sucrose and related enzyme activities (Additional file 1: Fig. S5).

#### Effects of Nano-Se on amino acid metabolism in melon leaves infected with *P. xanthii*

Amino acid homeostasis is interconnected with plant immune signaling pathways [63]. Relative to their controls, Nano-Se treatment had no discernable effect on total free amino acid contents of the four cultivars either prior to or following infection with powdery mildew (Additional file 1: Fig. S6A). However, Nano-Se treated plants of all cultivars showed a significantly higher content of glutamate of at 0 dpi (52–107%; B) and those of ZZX, HMC and JX also showed significant increases of 44–56% at 10 dpi. Nano-Se also promoted increases in glutamine synthase activities in all cultivars prior to infection (16–24%) and in JSG and JX at 10 dpi by 32% and 17%, respectively (C). Significant increases in leaf GABA content were observed in all cultivars tested prior to infection (21–88%) and at 10 dpi (50–199%) (D). The application of GABA was reported to increase plant tolerance to stress by regulating the expression of genes involved in plant signal transduction, hormone, ROS and polyamine metabolism [64]. Hydroxyproline levels were also significantly increased by Nano-Se in all four cultivars by 17–86% at 0 dpi and by 8–37% at 10 dpi (E). In the present study, the observed increase in amino acid content of melon leaf after Nano-Se treatment occurred with increases in the levels of glutamate, GABA and GS activity (Additional file 1: Fig. S6).

### Effects of Nano-Se on secondary metabolism

#### Polyamine metabolism

**Fig. 7:**
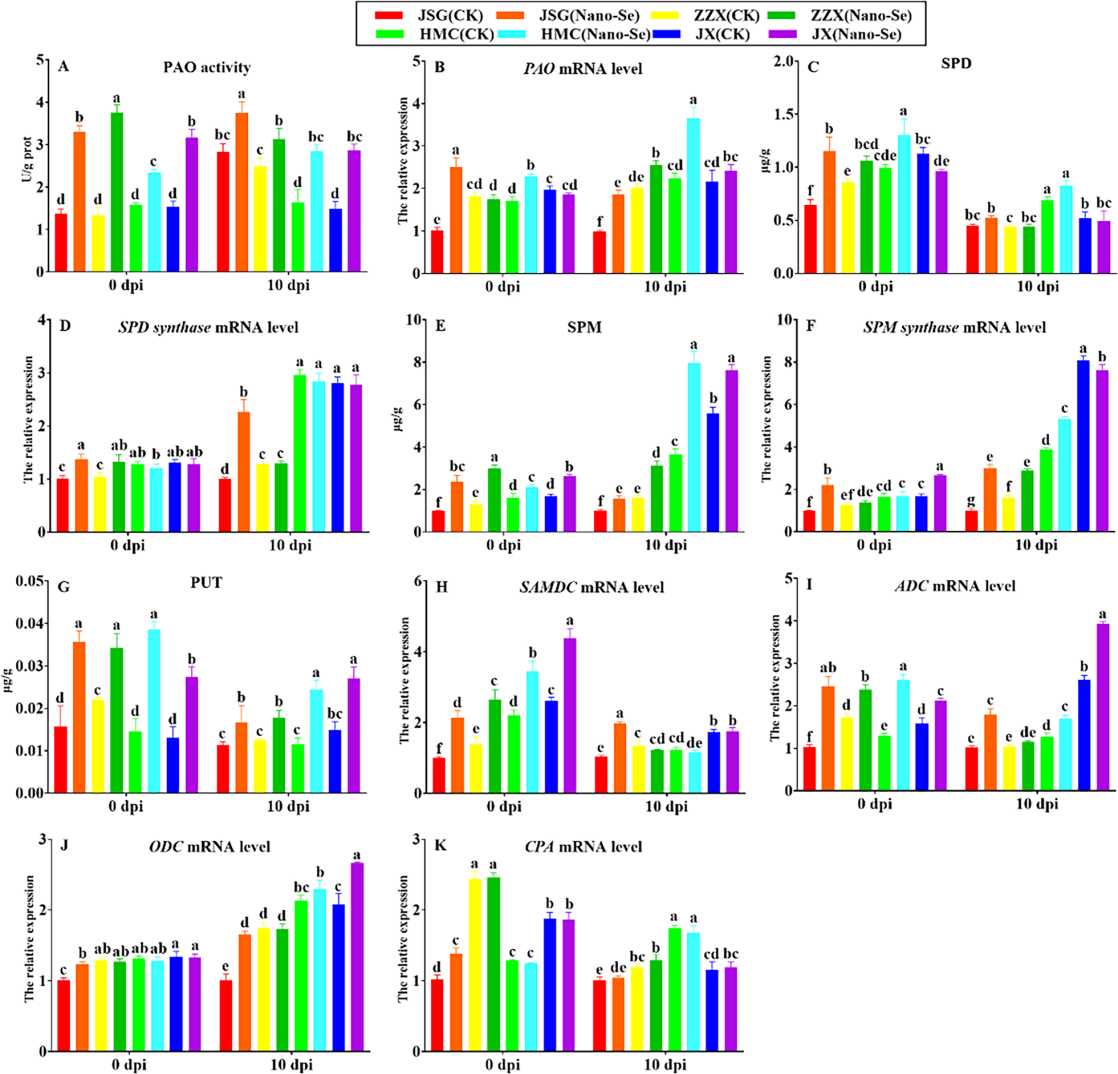
Effect of Nano-Se on leaf polyamine metabolism at 0 and 10 dpi. The figure layout, cultivars and conditions utilized are as in Fig. 1. *SAMDC*: *S-adenosylmethionine decarboxylase*;* ADC*: *arginine decarboxylase*; *ODC*: *ornithine decarboxylase*; *CPA*: *N-carbamyl putrescine hydrolase*

A possible role for free polyamines and the regulation of polyamine catabolism in plant resistance has been proposed [65]. The PAO activity in all cultivars was enhanced by the Nano-Se treatment at 0 and 10 dpi by 48–182% and 26–92%, respectively (Fig. 7A). These Nano-Se induced changes were accompanied by relative increases in *PAO* expression in JSG (150%) and HMC (34%) prior to inoculation and in all infected cultivars at 10 dpi by 12–86% (B). At 0 dpi, the Nano-Se treatment significantly increased SPD contents in JSG, ZZX and HMC by 78, 24 and 32%, respectively. However, at 10 dpi, the changes in SPD content of four cultivars were not significant (C). The Nano-Se treatment significantly increased *SPD synthase* mRNA levels in JSG, ZZX at 0 dpi by 36 and 27%, respectively (D).

Compared with the control group, Nano-Se treated plants showed significantly increased SPM levels in the four cultivars at 0 and 10 dpi by 30–139% and 36–118%, respectively (E). At 0 dpi, the Nano-Se treatment significantly increased *SPM synthase* mRNA levels in JSG and JX by 122 and 58%, respectively and at 10 dpi, by 200, 78 and 37% in the JSG, ZZX and HMC cultivars, respectively (F). The Nano-Se treatment increased PUT in the four cultivars at 0 and 10 dpi by 56–165% and 43–112%, respectively (G). Polyamines were also suggested to play an important role in environmental stress responses [66] and the metabolism of mainly putrescine, spermine and spermidine can affect the ability of fungi to cope with environmental challenges [67].

*S-adenosylmethionine decarboxylase* mRNA was increased in all four cultivars at 0 dpi by 57–115%, whereas at 10 dpi, only JSG showed an increase of 92% by Nano-Se (H). Except for ZZX, Nano-Se treatment increased *arginine decarboxylase* expression in three cultivars at 0 and 10 dpi by 34–140% and 34–76%, respectively (I). Conversely, no significant effects of Nano-Se treatment on the expression of *ornithine decarboxylase* and *N-carbamyl putrescine hydrolase* were observed (J, K). Conversely, the Nano-Se pretreatment potentiated PAO activity, the levels of SPD, SPM and PUT, as well as the mRNA levels of *PAO*, *SPMS*, *S-adenosylmethionine decarboxylase* and *arginine decarboxylase* (Fig. 7).

#### Effects of Nano-Se on melon leaf lignin metabolism

Additional file 1: Fig. S7 shows that Nano-Se promoted increases in lignin content in all cultivars at both 0 and 10 dpi (17–49%; A). The accumulation of phenylpropanoid-derived secondary metabolites, such as phenolic acids, flavonoids, and lignin have been associated with citric plant defense against fungi [68]. In our study, the improvement of melon resistance to powdery mildew disease by Nano-Se was also shown to enhance lignin synthesis (Additional file 1: Fig. S7). In agreement, the accumulation of lignin was shown to be linked to the improvement of antifungal activity and the defensive response in citrus fruits [68]. Conversely, Nano-Se had no significant effect on leaf CAD activity or *CAD* and *CCR* mRNA levels in the four cultivars at 0 or 10 dpi (B, C, D).

#### Alterations in phenylpropanoid metabolism in response to *P. xanthii* infection and Nano-Se treatment

**Fig. 8:**
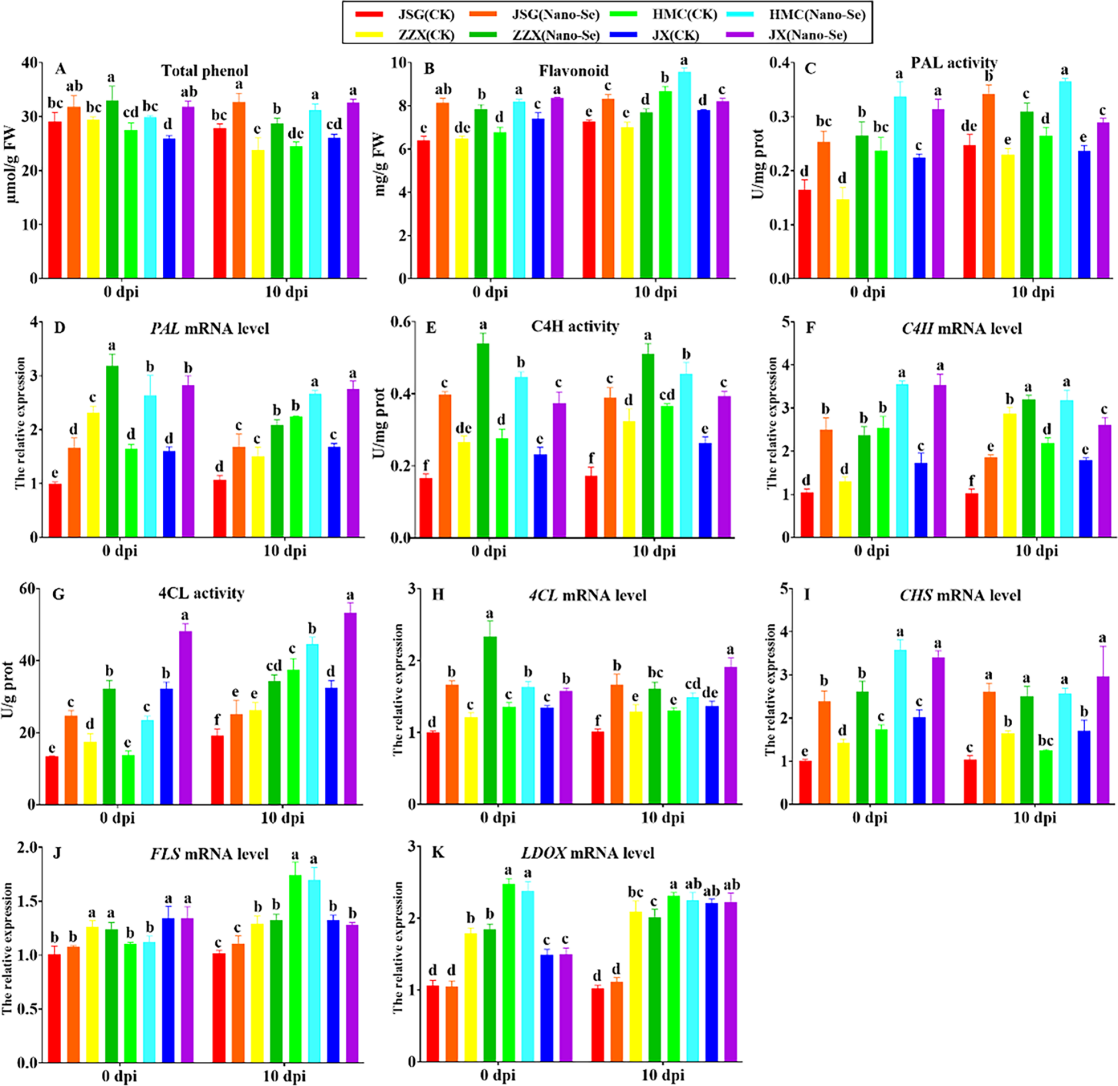
Effect of Nano-Se on leaf phenylpropanoid metabolism at 0 and 10 dpi. The figure layout, cultivars and conditions utilized are as in Fig. 1

The influence of Nano-Se treatment groups on phenylpropanoid metabolism in melon leaves at different of *P. xanthii* infection is depicted in Fig. 8. The Nano-Se treatment was seen to significantly increase the total phenols in ZZX and JX cultivars at 0 dpi by 12–22%, respectively, whereas at 10 dpi, Nano-Se treatment groups significantly increased the total phenol content in all four cultivars by 17–27%, respectively (A). The flavonoid contents in all Nano-Se plants were significantly increased at 0 and 10 dpi by 13–27% and 5–15%, respectively (B). The Nano-Se treatment increased PAL activity in the four cultivars by 40–80% at 0 dpi and by 22–38% at 10 dpi (C). This was accompanied by significant increases in *PAL* mRNA levels of in all four cultivars at 0 and 10 dpi by 37–76% and 18–64%, respectively (D). The flavonoids and phenolic acids were shown linked to the improvement of antifungal activity and the defensive response in citrus fruits [68]. Increases in PAL activity, flavonoid and total phenol contents contribute to plant disease resistance [69].

Nano-Se treatment also increased C4H activity in the four cultivars at 0 and 10 dpi by 61–140% and 24–126%, respectively (E), which were similarly accompanied by respective increases at the mRNA level of 40–141% and 12–80% (F). Before infection (0 dpi), Nano-Se treatment increased the 4CL activity (50–84%) in four cultivars. At 10 dpi, Nano-Se treatment significantly increased the 4CL activity of the four cultivars by 19–64% (G). Nano-Se treatment groups significantly increased *4CL* mRNA levels of all four cultivars at 0, 10 dpi by 18–67% and 14–65%, respectively (H). The Nano-Se treatment also increased the *CHS* mRNA levels in the four cultivars by 69–135% at 0 dpi and by 52–150% at 10 dpi (I). The Nano-Se treatment had no significant effect on the mRNA levels of *FLS* or *LDOX* in any cultivar (J, K). In our study, the improvement of melon resistance to powdery mildew disease by Nano-Se was shown to enhance phenylpropanoid metabolism (Fig. 8).

### RNA-Seq analysis of Nano-Se effects on melon leaf gene expression in uninfected and *P. xanthii* infected seedlings

**Fig. 9:**
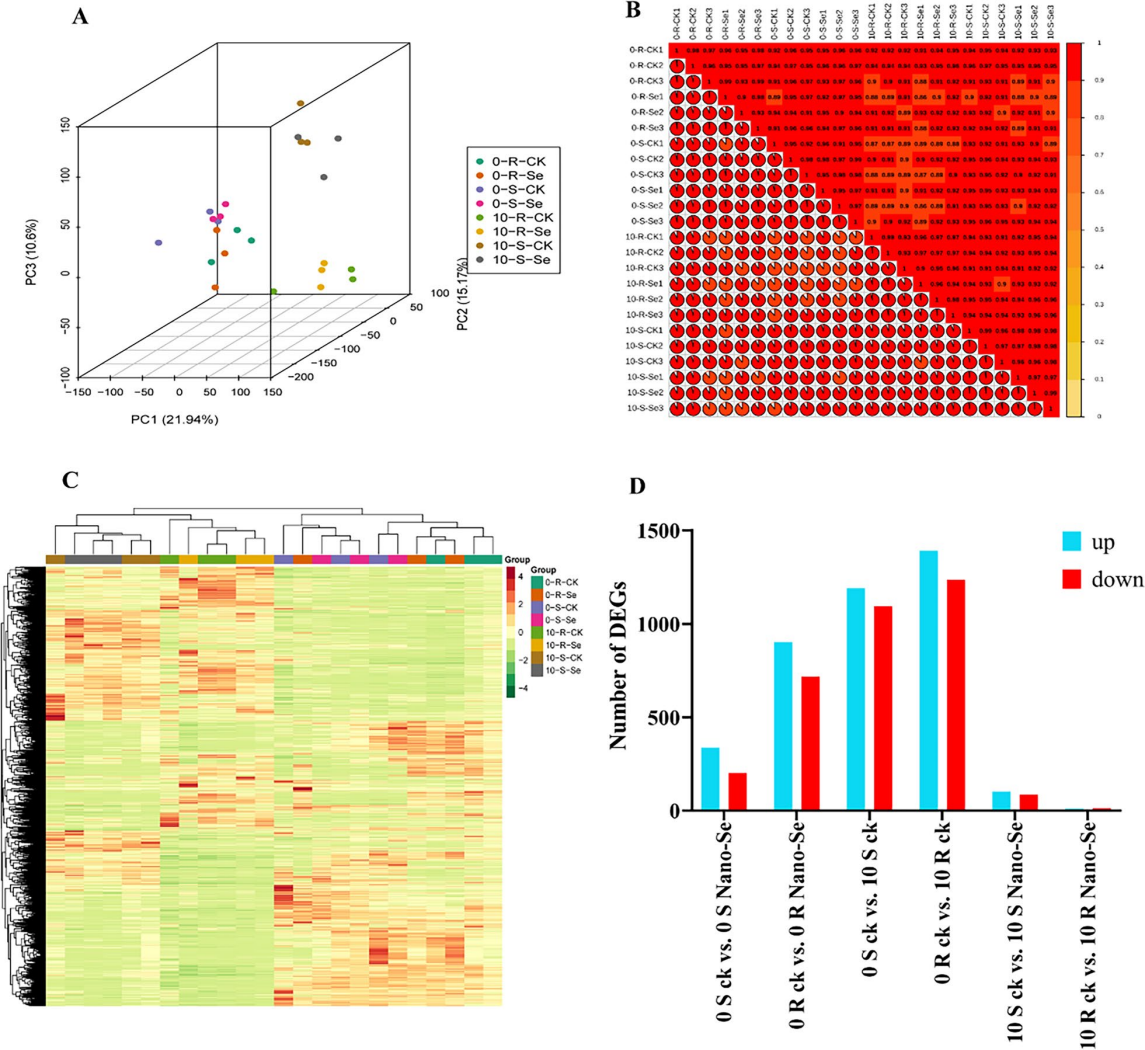
A general transcriptomic analysis of the melon seedling response to powdery mildew infected with or without Nano-Se pretreatment. (**A**) Principal component analysis, (**B**) Correlation analysis, (**C**) Differential gene cluster analysis and (**D**) Differential gene analysis. The S and R represent the susceptible cultivar JSG and resistant cultivar HMC at 0 and 10 dpi, respectively. CK, Nano-Se represent the control leaves and those sprayed with 5.0 mg⋅L^−1^ Nano-Se, respectively

A PCA analysis of leaf RNA-Seq data was conducted with the samples of the susceptible and resistant cultivars at 0 and 10 dpi, with and without Nano-Se pretreatment. The PCA readily discriminated the 0 dpi and 10 dpi treatments, with PC1, PC2 and PC3 explaining 21.9%, 15.2% and 10.6% of the total variance, respectively. The melon leaf samples at 0 dpi and 10 dpi, as well as the control and Nano-Se treated plants were clearly distinguished (Fig. 9A). Based on the correlation heat map analysis of FPKM values, it was found that there was a good correlation between the three biological replicates (Fig. 9B). A total of 1,110,261,430 clean reads and 166.56 G clean bases were obtained in all samples (Additional file 2: Table S5). As shown in the visualization of the heat map, obvious hierarchical clustering of different types of samples could be seen (Fig. 9C). Prior to infection (0 dpi), the numbers of DEGs detected in response to the Nano-Se treatment in the susceptible (S; JSG) and resistant (R; HMC) cultivars were 539 (338 up- and 201 down-regulated, Additional file 1: Fig S8A) and 1621 (902 up- and 719 down-regulated, Additional file 1: Fig S8B), respectively. In response to *P. xanthii* infection (10 dpi), a larger number of DEGs were detected in seedlings untreated with Nano-Se, where 2285 (1191 up- and 1094 down-regulated) were detected in the susceptible cultivar JSG (Additional file 1: Fig. S8C), and 2629 (1392 up- and 1237 down-regulated) were detected in the resistant cultivar, HMC (Additional file 1: Fig. S8D). Conversely, much fewer DEGs were detected in infected seedlings that had been subjected to the Nano-Se pretreatment, where 188 (102 up- and 86 down-regulated), and 23 (10 up- and 13 down-regulated) were detected in the susceptible and resistant cultivars, respectively. These results are summarized in a bar chart in Fig. 9D.

The functions of the DEGs identified were annotated from matched identifiers from 7 databases (KEGG, KOG, NR, Pfam, Swissprot, Tremble, and GO) (Additional file 2: Table S6). The biological functions of DEGs were subjected to GO enrichment analysis. In the comparison of control and Nano-Se treatment groups at 0 dpi, the main enriched terms indicated alterations in catalytic activity, DNA polymerase activity, monooxygenase activity, nucleotidyl transferase activity, RNA-directed DNA polymerase activity, transferase activity, chloroplast thylakoid, plastid thylakoid, coenzyme and tetrapyrrole binding. Between the conditions of 0 and 10 dpi, both R and S cultivars mainly showed enrichment in the terms chloroplast/thylakoid, photosynthetic membrane, plastid thylakoid, and thylakoid part (Additional file 1: Fig S9). The main differentially enriched terms between the control and Nano-Se treatment group at 10 dpi were bacterium and drug, secondary metabolic and biosynthetic process and tetrapyrrole binding (Additional file 1: Fig S10).

**Fig. 10:**
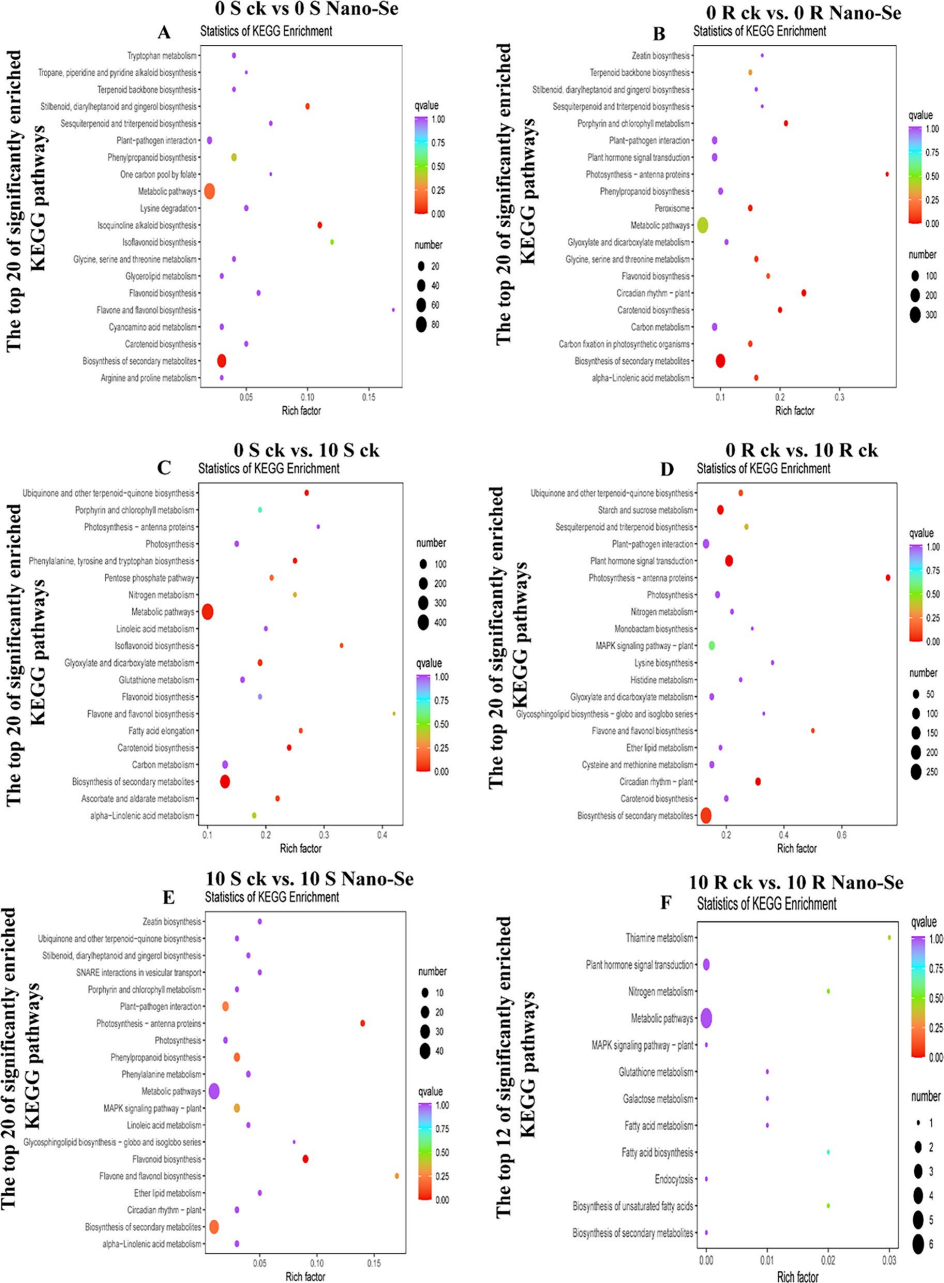
KEGG enrichment analysis of the seedling leaf DEGs after powdery mildew infection with or without prior Nano-Se treatment. **A**: 0 S ck *vs* 0 S Nano-Se, **B**: 0 R ck *vs* 0 R Nano-Se, **C**: 0 S ck *vs* 10 S ck, **D**: 0 R ck *vs* 10 R ck, **E**: 10 S ck *vs* 10 S Nano-Se, **F**: 10 R ck *vs* 10 R Nano-Se. The Y-axis represents the enriched KEGG pathway, and the X-axis represents the rich factor. The dot size represents the number of DEGs in the pathway, and the dot color represents the q-value

KEGG enrichment analysis was also performed. The DEGs obtained from the comparison groups of 0 S ck *vs.* 0 S Nano-Se, 0 R ck *vs.* 0 R Nano-Se, 0 S ck *vs.* 10 S ck, 0 R ck *vs*. 10 R ck, 10 S ck *vs.* 10 S Nano-Se, and 10 R ck *vs.* 10 R Nano-Se were annotated on 78, 115, 123, 125, 64, and 126 KEGG pathways, respectively (Additional file 2: Tables S7–S12), of which the 20 most significantly enriched pathways in each comparison are shown in Fig. 10. In particular, the biosynthesis of secondary metabolites (ko01110) was significantly enriched in four comparisons (0 S ck *vs.* 0 S Nano-Se, 0 R ck *vs.* 0 R Nano-Se, 0 S ck *vs.* 10 S ck, and 0 R ck *vs.* 10 R ck). The flavonoid biosynthesis (ko00941; 0 R ck *vs.* 0 R Nano-Se), isoflavonoid biosynthesis (ko00943; 0 S ck *vs.* 10 S ck) and flavone and flavonol biosynthesis (ko00944; 0 R ck *vs.* 10 R ck) were significantly enriched in two cultivars. KEGG pathway analysis of tea plants treated with Na_2_SeO_3_ and Na_2_SeO_4_ revealed that many genes involved in amino acid and glutathione metabolism were up-regulated, and genes and proteins associated with glutathione metabolism and biosynthesis of ubiquinone and terpenoid were highly expressed [70]. The DEGs associated with the biosynthesis pathway of catechins, caffeine and theanine in tea were affected by Nano-Se [71].

Therefore, the biosynthesis of secondary metabolites, including flavonoids and isoflavonoids might play an important role in the enhanced resistance to powdery mildew infection displayed by Nano-Se treated plants. In addition, flavone and flavonol biosynthesis (ko00944) were also enriched in the process of Nano-Se inducing resistance to *P. xanthii* infection. After Na_2_SeO_3_ treatment, genes related to phenylpropanoid, flavonoid and anthocyanin biosynthesis were significantly up-regulated, resulting in the accumulation of anthocyanin metabolites in grain wheat [72], which is similar to the results of this study. The beneficial effects of Nano-Se against melon powdery mildew therefore appears to be achieved through its effects on multiple pathways. A list of the 18 most affected DEGs, including genes for two antioxidant enzyme genes, nine phenylpropanoid pathway genes, two flavonoid synthesis pathway genes and five glycosyl transferase genes, is provided in Additional file 2: Table S13 as genes worthy of further investigation. The effects of Nano-Se appear to be most evident prior to mildew infection, indicating that Nano-Se mostly affects melon basal resistance, rather than reinforcing the plant response to infection.

The concentration of Nano-Se in this study was 5.0 mg·L^−1^. We verified that the prepared Nano-Se were not toxic to plants. Melon seedlings at different stages of treatment with Nano-Se are shown in Additional file 1: Fig S11. Low doses of selenium improved plant stress resistance through photosynthesis, while high doses of selenium interfered with nitrogen assimilation, resulting in decreased nitrogen compound synthesis ability [73]. In this study, the levels of glutamate, GS and GABA associated with the nitrogen cycle increased after Nano-Se treatment, which further indicated that 5 mg·L^−1^ Nano-Se is not toxic to melon. Low concentrations (20 μM) of selenates were also shown to be beneficial for plant growth, which was related to the antioxidant effect of selenium [74].

## Conclusion

The foliar application 5.0 mg·L^−1^ Nano-Se to melon seedlings reduced the incidence and disease index of *P. xanthii* powdery mildew disease. Nano-Se was seen to enhance ROS scavenging, polyamine levels, phenylpropanoid metabolism and plant hormone signal transduction and that these most of these effects were significant in the pre-infection stages. Transcriptome analysis revealed that the alterations in the biosynthesis of secondary metabolites, including flavonoid, isoflavonoids, flavone and flavonol biosynthesis might play essential roles in enhancing resistance to powdery mildew infection by Nano-Se treatment. The data suggests that the Nano-Se pretreatment largely enhances the basal resistance of melon to *P. xanthii* through its effects on multiple secondary metabolite and functional pathways.

### Supplementary Information

**Additional file 1:** **Figure S1. **Characterization results of Nano-Se. **A** Visual aspect of colloidal Nano-Se. **B**, **C** SEM images of Nano-Se under different magnification. **D** SEM/EDX images of Nano-Se. Scale bar, 0.5 μm. **E** Distribution of total spectrum diagram. **F** Energy-dispersive X-ray (EDX) spectroscopy spectrum of Nano-Se. **G** TEM images of Nano-Se. Scale bar, 0.05 μm. TEM/EDX images of C (**H**), O (**I**) and Se (**J**). **K** AFM image of Nano-Se. **L** Size distribution by intensity of Nano-Se. **M** Particle size distribution of Nano-Se by DLS. **N** XRD patterns of Nano-Se. **O** FTIR spectra of Nano-Se. **Figure S2.** Scanning electron microscopic (SEM) of control and Nano-Se treated melon leaves at different infection stages of powdery mildew infection. The days after infection (dpi) are indicted above the panels. The cultivars used (JSG and ZZX) are indicated to the left. The upper two and lower two panel rows represent the control and treated leaves (5.0 mg L^−1^), respectively. **Figure S3.** Effects of Nano-Se on leaf lipoxygenase activity and mRNA levels in melon cultivars of different resistances to powdery mildew at 0 and 10 dpi. JSG, ZZX, HMC and JX represent the four melon cultivars *Jia shi*, *Zao zui xian* (susceptible) and *Huang meng cui*, *Jun xiu* (resistant), respectively. CK and Nano-Se represented the control leaves and those sprayed with 5.0 mg⋅L^−1^ Nano-Se, respectively. dpi = days post inoculation. Different letters indicate a significant difference (*p* < 0.05) between the treatments. The error bars represent standard deviations (n = 4). **Figure S4.** Effects of Nano-Se on leaf plant hormone content in the four melon cultivars at 0 and 10 dpi. The figure layout, cultivars and conditions utilized are as in Fig. S3. **Figure S5.** Effects of Nano-Se on leaf carbohydrate metabolism in the four melon cultivars at 0 and 10 dpi. The figure layout, cultivars and conditions utilized are as in Fig. S3. **Figure S6.** Effects of Nano-Se on leaf amino acid content in the four melon cultivars at 0 and 10 dpi. The figure layout, cultivars and conditions utilized are as in Fig. S3. **Figure S7.** Effect of Nano-Se on leaf lignin synthesis in the four melon cultivars at 0 and 10 dpi. The figure layout, cultivars and conditions utilized are as in Fig. S3. **Figure S8.** Volcano plot of differential gene expression between resistant (R) and susceptible (S) cultivars and their response to powdery mildew infection (0, 10 dpi) with Nano-Se pretreatment (Nano-Se) or without (ck). Seedling leaves were used in the comparisons. **A** 0 S ck *vs* 0 S Nano-Se, **B** 0 R ck *vs* 0 R Nano-Se, **C** 0 S ck *vs* 0 S ck, **D** 0 R ck *vs* 10 R ck, **E** 10 S ck *vs* 10 S Nano-Se, **F** 10 R ck *vs* 10 R Nano-Se. **Figure S9.** GO enrichment analysis of leaf DEGs between susceptible and resistant cultivars and their response to powdery mildew infection with and without the Nano-Se pretreatment. Seedling leaves were used in the comparisons. **A** 0 S ck *vs* 0 S Nano-Se, **B** 0 R ck *vs* 0 R Nano-Se, **C** 0 S ck *vs* 0 S ck, **D** 0 R ck *vs* 10 R ck, **E** 10 S ck *vs* 10 S Nano-Se, **F** 10 R ck *vs* 10 R Nano-Se. **Figure S10.** GO enrichment histogram of DEGs in response to the Nano-Se pretreatment in powdery mildew infected (10 dpi) leaves of the susceptible (S) and resistant (R) cultivars. The X-axis represents the ratio of the GO annotation of the DEGs to the total number GO annotation for all genes. The Y-axis represents the GO entry term. The label to the right of the graph represents the category to which the GO entry belongs. A: 10 S ck *vs* 10 S Nano-Se, B: 10 R ck *vs* 10 R Nano-Se. **Figure S11.** Melon seedlings of four different cultivars at different stages of treatment with Nano-Se. The days after infection (dpi) are indicted above the panels. The cultivars used (JSG, ZZX, HMC, and JX) are indicated to the left. The labels to the right indicate the rows of control and treated leaves (5.0 mg L^−1^), respectively.

**Additional file 2: Table S1.** UPLC-MS/MS conditions for the detection of JA, SA, IAA and polyamine. **Table S2.** Primers used for quantitative real time PCR of selected genes involved in secondary metabolism. **Table S3.** Effects of Nano-Se on stem weights of melon cultivars of with different resistances to powdery mildew. **Table S4.** Anatomical structural characteristics of melon leaves inoculated with *P*. *xanthii* (1 and 10 dpi) with or without Nano-Se pretreatment. **Table S5.** Statistics of the RNA-Seq data generated by the Illumina HiSeq Platform in the study. **Table S6.** Functional annotations on the genes against public database. **Table S7.** KEGG enrichment analysis of DEGs in the 0 S ck vs. 0 S Nano-Se. **Table S8.** KEGG enrichment analysis of DEGs in the 0 R ck vs. 0 R Nano-Se. **Table S9.** KEGG enrichment analysis of DEGs in the 0 S ck vs. 10 S ck. **Table S10.** KEGG enrichment analysis of DEGs in the 0 R ck vs. 10 R ck. **Table S11.** KEGG enrichment analysis of DEGs in the 10 S ck vs. 10 S Nano-Se. **Table S12.** KEGG enrichment analysis of DEGs in the 10 R ck vs. 10 R Nano-Se. **Table S13.** FPKM values of candidate DEGs involved in the secondary metabolism biosynthetic pathway during *P. xanthii* powdery mildew infection.

## Data Availability

All data generated or analysed during this study are included in this published article.
