# Supplementary material for: Nano-selenium enhances melon resistance to Podosphaera xanthii by enhancing the antioxidant capacity and promoting alterations in the polyamine, phenylpropanoid and hormone signaling pathways
Source: J Nanobiotechnology. 2023 Oct 16;21:377. doi: 10.1186/s12951-023-02148-y (PMC10577987; doi:10.1186/s12951-023-02148-y)
Supplement: Supplementary file 1 — Additional file 1: Figure S1. Characterization results of Nano-Se. A Visual aspect of colloidal Nano-Se. B, C SEM images of Nano-Se under different magnification. D SEM/EDX images of Nano-Se. Scale bar, 0.5 μm. E Distribution of total spectrum diagram. F Energy-dispersive X-ray (EDX) spectroscopy spectrum of Nano-Se. G TEM images of Nano-Se. Scale bar, 0.05 μm. TEM/EDX images of C (H), O (I) and Se (J). K AFM image of Nano-Se. L Size distribution by intensity of Nano-Se. M Particle size distribution of Nano-Se by DLS. N XRD patterns of Nano-Se. O FTIR spectra of Nano-Se. Figure S2. Scanning electron microscopic (SEM) of control and Nano-Se treated melon leaves at different infection stages of powdery mildew infection. The days after infection (dpi) are indicted above the panels. The cultivars used (JSG and ZZX) are indicated to the left. The upper two and lower two panel rows represent the control and treated leaves (5.0 mg L−1), respectively. Figure S3. Effects of Nano-Se on leaf lipoxygenase activity and mRNA levels in melon cultivars of different resistances to powdery mildew at 0 and 10 dpi. JSG, ZZX, HMC and JX represent the four melon cultivars Jia shi, Zao zui xian (susceptible) and Huang meng cui, Jun xiu (resistant), respectively. CK and Nano-Se represented the control leaves and those sprayed with 5.0 mg⋅L−1 Nano-Se, respectively. dpi = days post inoculation. Different letters indicate a significant difference (p < 0.05) between the treatments. The error bars represent standard deviations (n = 4). Figure S4. Effects of Nano-Se on leaf plant hormone content in the four melon cultivars at 0 and 10 dpi. The figure layout, cultivars and conditions utilized are as in Fig. S3. Figure S5. Effects of Nano-Se on leaf carbohydrate metabolism in the four melon cultivars at 0 and 10 dpi. The figure layout, cultivars and conditions utilized are as in Fig. S3. Figure S6. Effects of Nano-Se on leaf amino acid content in the four melon cultivars at 0 and 10 dpi. The figur [file 12951_2023_2148_MOESM1_ESM.docx]

**Supporting information**

Nano-selenium enhances melon resistance to *Podosphaera xanthii* by promoting the antioxidant ability and alteration in the polyamine, phenylpropanoid and hormone signaling pathways

Lu Kang^a,b^, Yangliu Wu^a,c^, Yujiao Jia^a^, Zhendong Chen^d^, Dexian Kang^d^ , Li Zhang^d^, Canping Pan^a*^

^a^ Key Laboratory of National Forestry and Grassland Administration on Pest Chemical Control & Innovation Center of Pesticide Research, College of Science, China Agricultural University, Beijing, 100193, China

^b^ Institute of Agricultural Quality Standards and Testing Technology, Xinjiang Academy of Agricultural Sciences, Urumqi 830091, China

^c^ School of Biological Science and Technology, University of Jinan, Jinan 250022, China

^d^ Vegetable Research Institute, Guangxi Zhuang Autonomous Region Academy of Agricultural Sciences, Nanning, 530000, China

**Corresponding author:** Canping Pan ^*^**E-mail:** [canpingp@cau.edu.cn](mailto:canpingp@cau.edu.cn)

**Fax:** +86-10-62733620; Tele: +86-10-62731978

**Address:** 2 Yuanmingyuan Western Road, Haidian District, Beijing 100193, China.


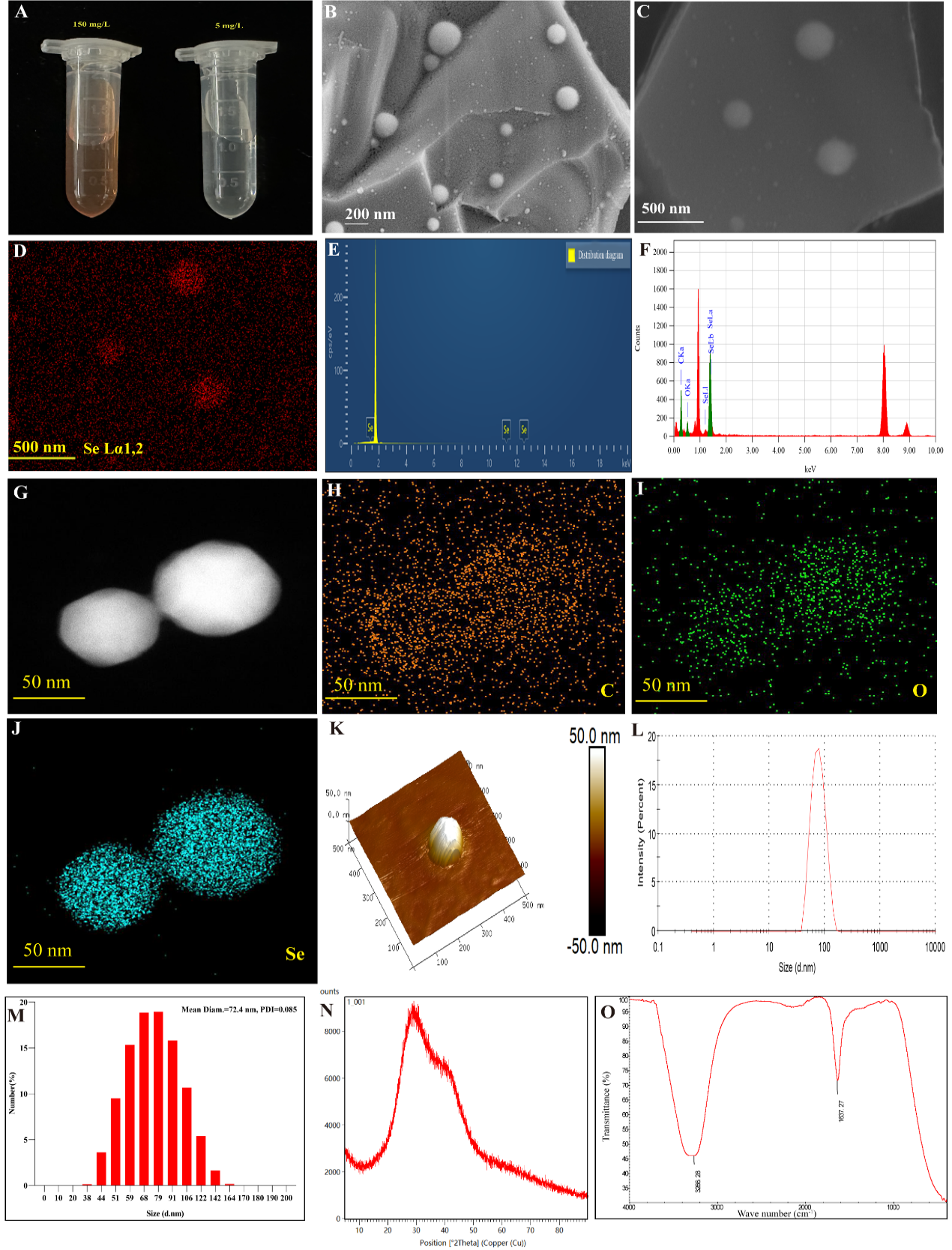


# Fig. S1. Characterization results of Nano-Se. (A) Visual aspect of colloidal Nano-Se. (B, C) SEM images of Nano-Se under different magnification. (D) SEM/EDX images of Nano-Se. Scale bar, 0.5 μm. (E) Distribution of total spectrum diagram. (F) Energy-dispersive X-ray (EDX) spectroscopy spectrum of Nano-Se. (G) TEM images of Nano-Se. Scale bar, 0.05 μm. TEM/EDX images of C (H), O (I) and Se (J). (K) AFM image of Nano-Se. (L) Size distribution by intensity of Nano-Se. (M) Particle size distribution of Nano-Se by DLS. (N) XRD patterns of Nano-Se. (O) FTIR spectra of Nano-Se.


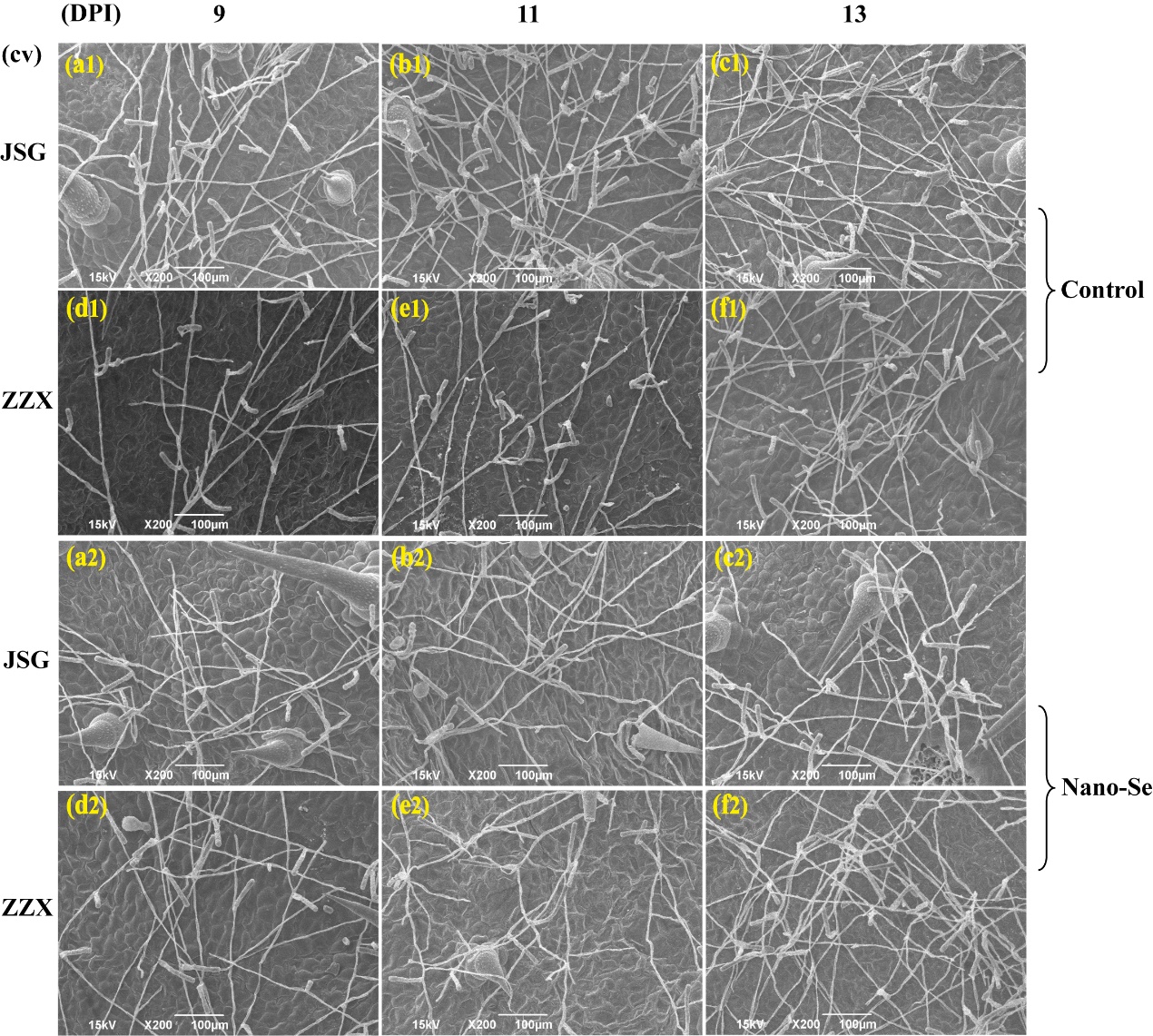


# Fig. S2. Scanning electron microscopic (SEM) of control and Nano-Se treated melon leaves at different infection stages of powdery mildew infection. The days after infection (dpi) are indicted above the panels. The cultivars used (JSG and ZZX) are indicated to the left. The upper two and lower two panel rows represent the control and treated leaves (5.0 mg L^-1^), respectively.


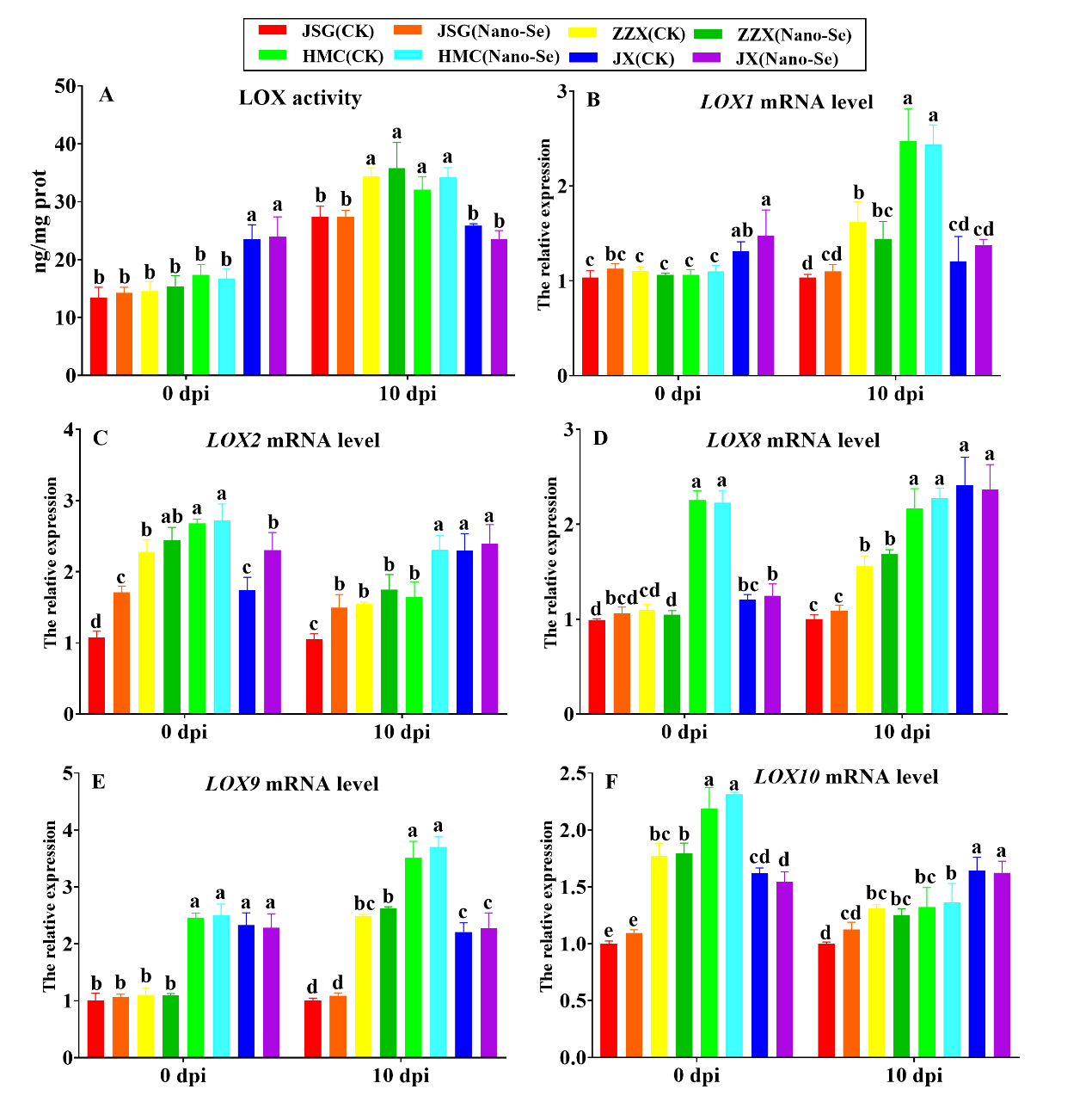


# Fig. S3. Effects of Nano-Se on leaf lipoxygenase activity and mRNA levels in melon cultivars of different resistances to powdery mildew at 0 and 10 dpi. JSG, ZZX, HMC and JX represent the four melon cultivars *Jia shi*, *Zao zui xian* (susceptible) and *Huang meng cui*, *Jun xiu* (resistant), respectively. CK and Nano-Se represented the control leaves and those sprayed with 5.0 mg⋅L^−1^ Nano-Se, respectively. dpi = days post inoculation. Different letters indicate a significant difference (*p* < 0.05) between the treatments. The error bars represent standard deviations (n = 4).

#
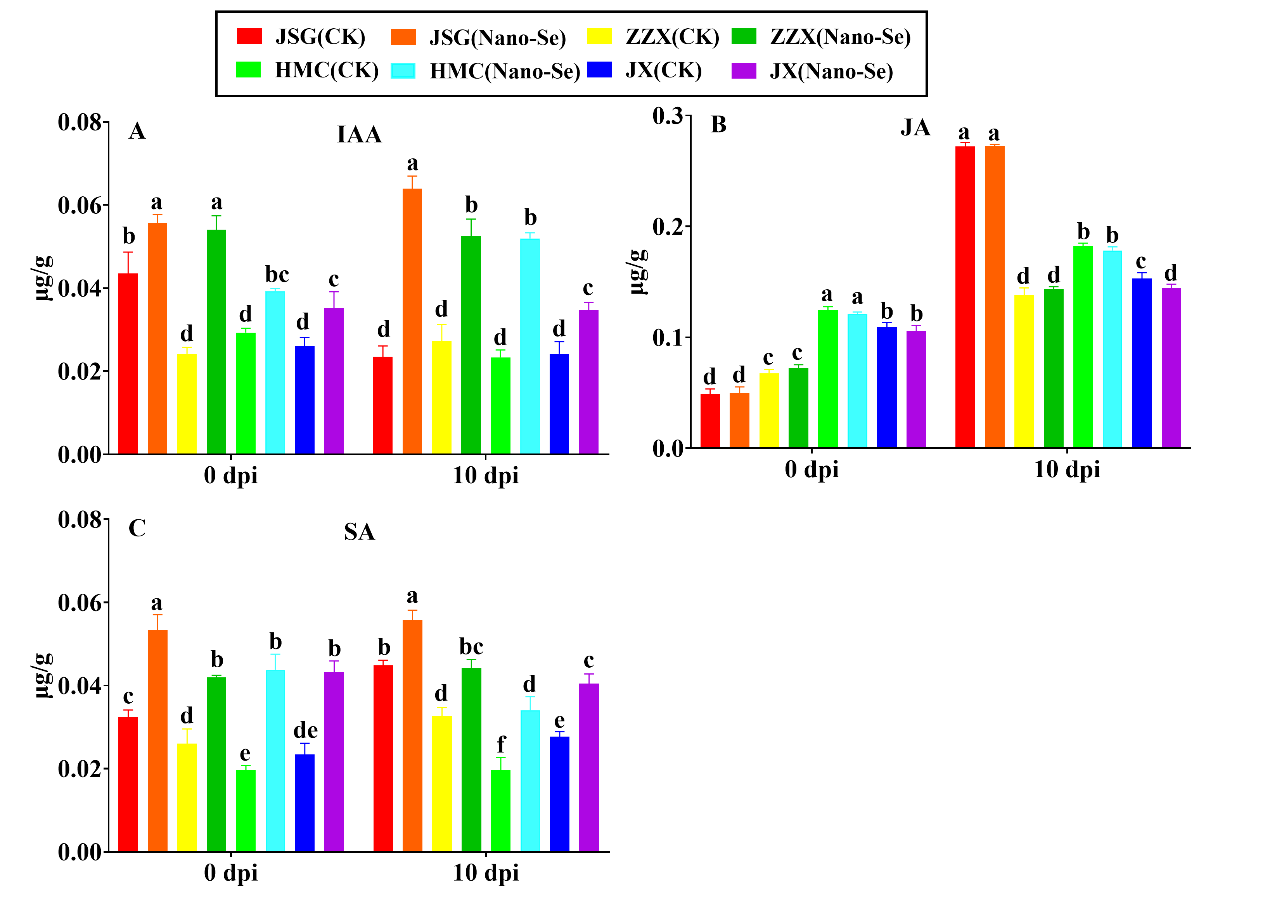
Fig. S4. Effects of Nano-Se on leaf plant hormone content in the four melon cultivars at 0 and 10 dpi. The figure layout, cultivars and conditions utilized are as in Fig. S3.


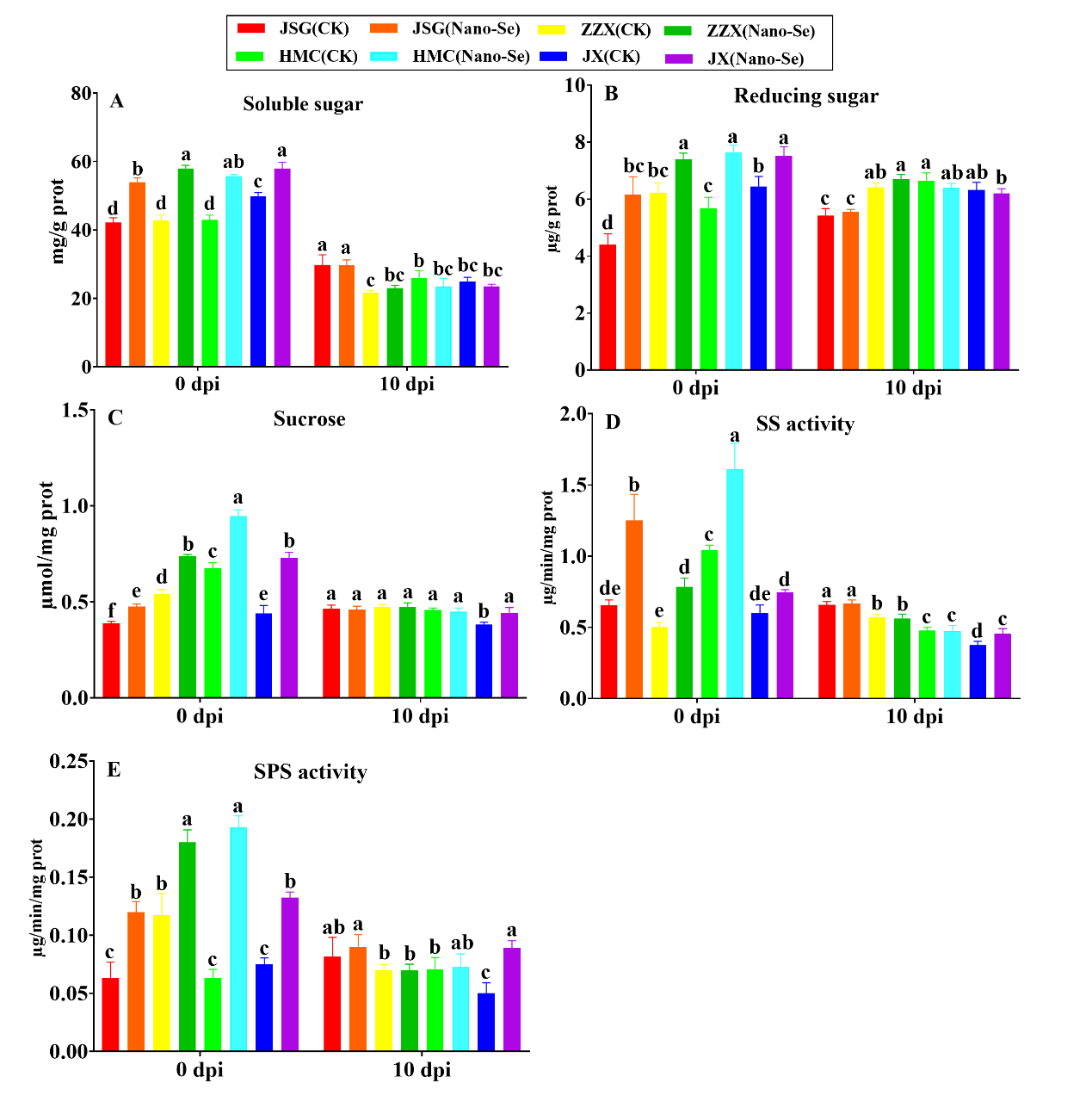


# Fig. S5. Effects of Nano-Se on leaf carbohydrate metabolism in the four melon cultivars at 0 and 10 dpi. The figure layout, cultivars and conditions utilized are as in Fig. S3.


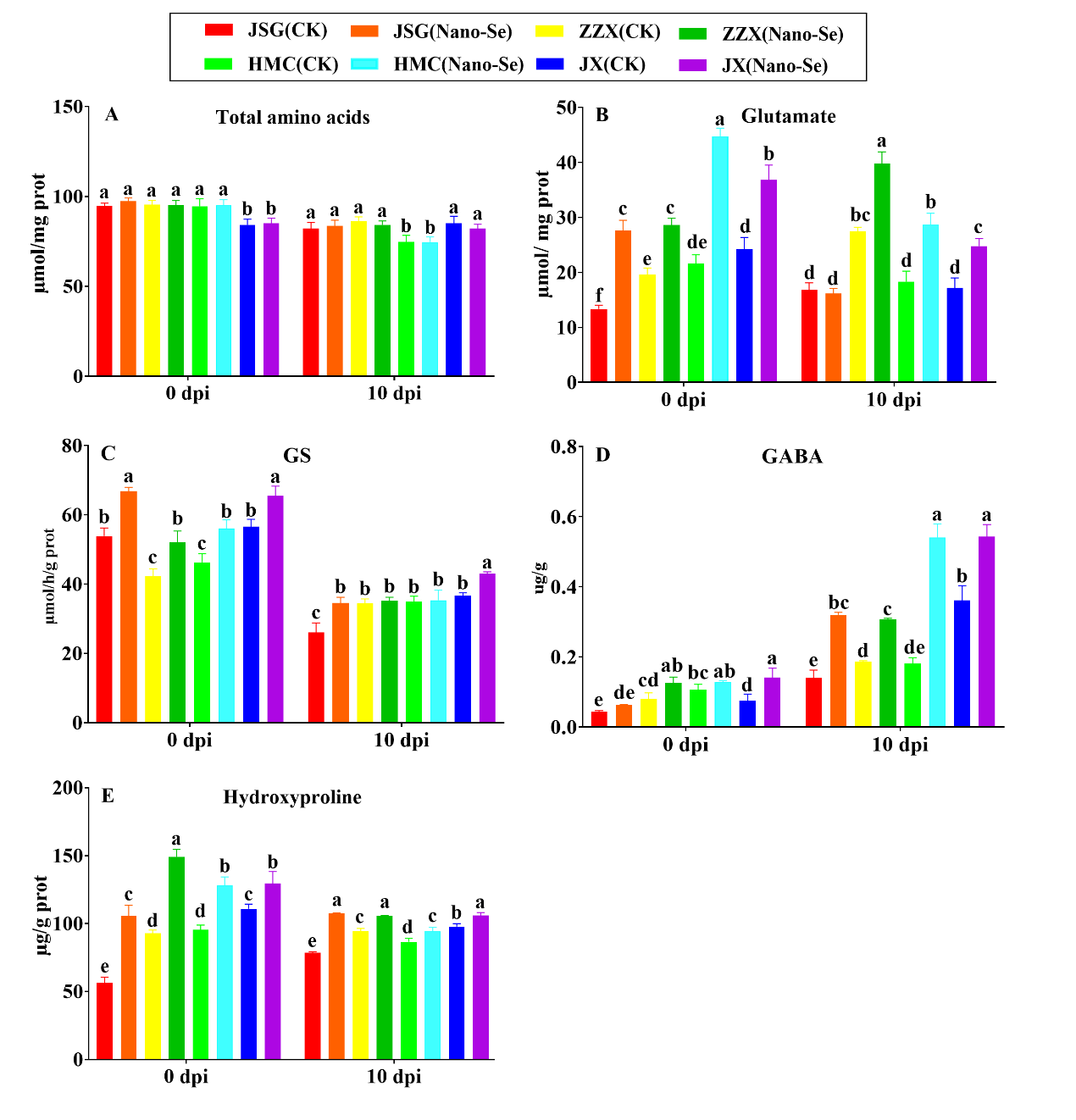


# Fig. S6. Effects of Nano-Se on leaf amino acid content in the four melon cultivars at 0 and 10 dpi. The figure layout, cultivars and conditions utilized are as in Fig. S3.


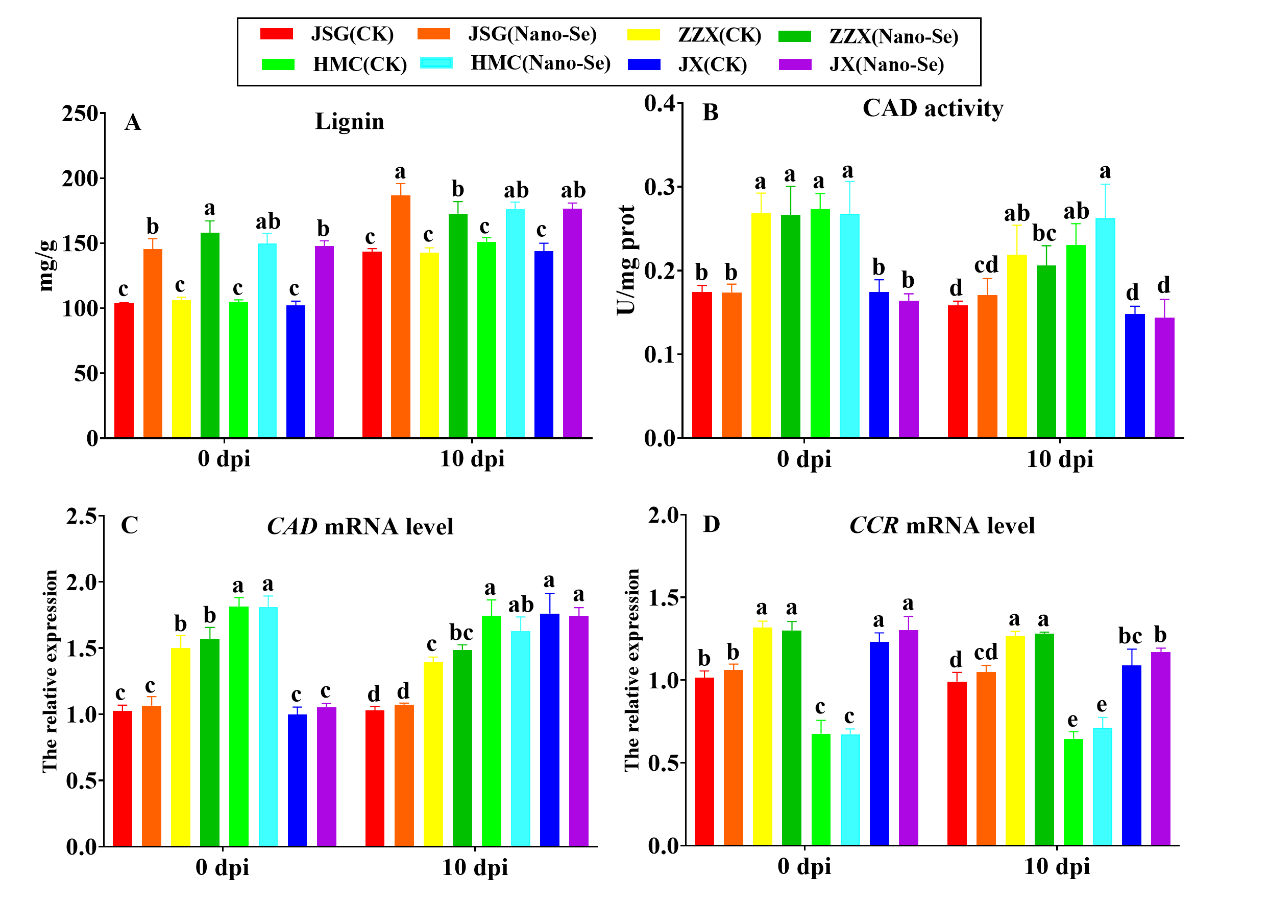


# Fig. S7. Effect of Nano-Se on leaf lignin synthesis in the four melon cultivars at 0 and 10 dpi. The figure layout, cultivars and conditions utilized are as in Fig. S3.

#
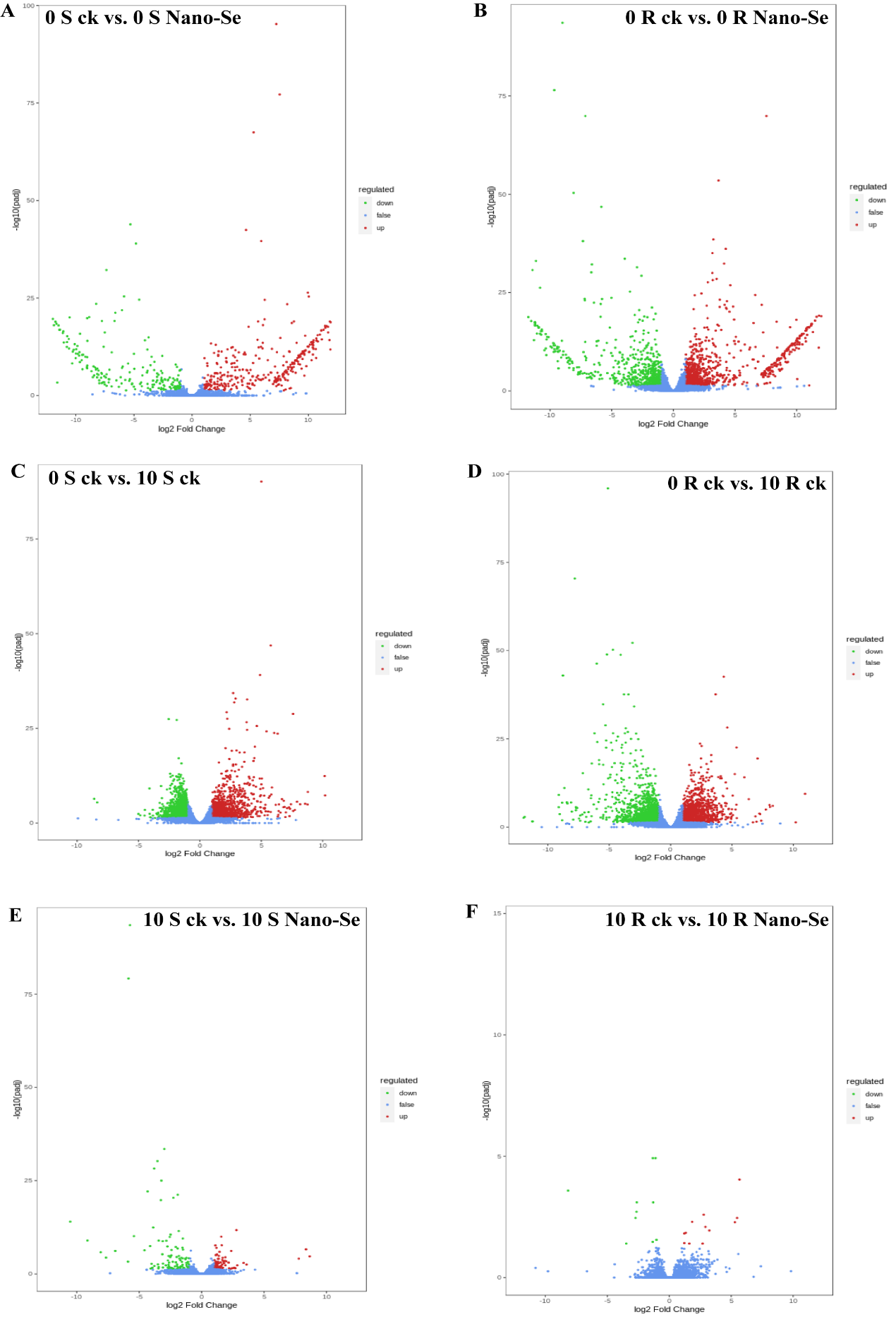
Fig. S8. Volcano plot of differential gene expression between resistant (R) and susceptible (S) cultivars and their response to powdery mildew infection (0, 10 dpi) with Nano-Se pretreatment (Nano-Se) or without (ck). Seedling leaves were used in the comparisons. A: 0 S ck *vs* 0 S Nano-Se, B: 0 R ck *vs* 0 R Nano-Se, C: 0 S ck *vs* 0 S ck, D: 0 R ck *vs* 10 R ck, E: 10 S ck *vs* 10 S Nano-Se, F: 10 R ck *vs* 10 R Nano-Se.

#
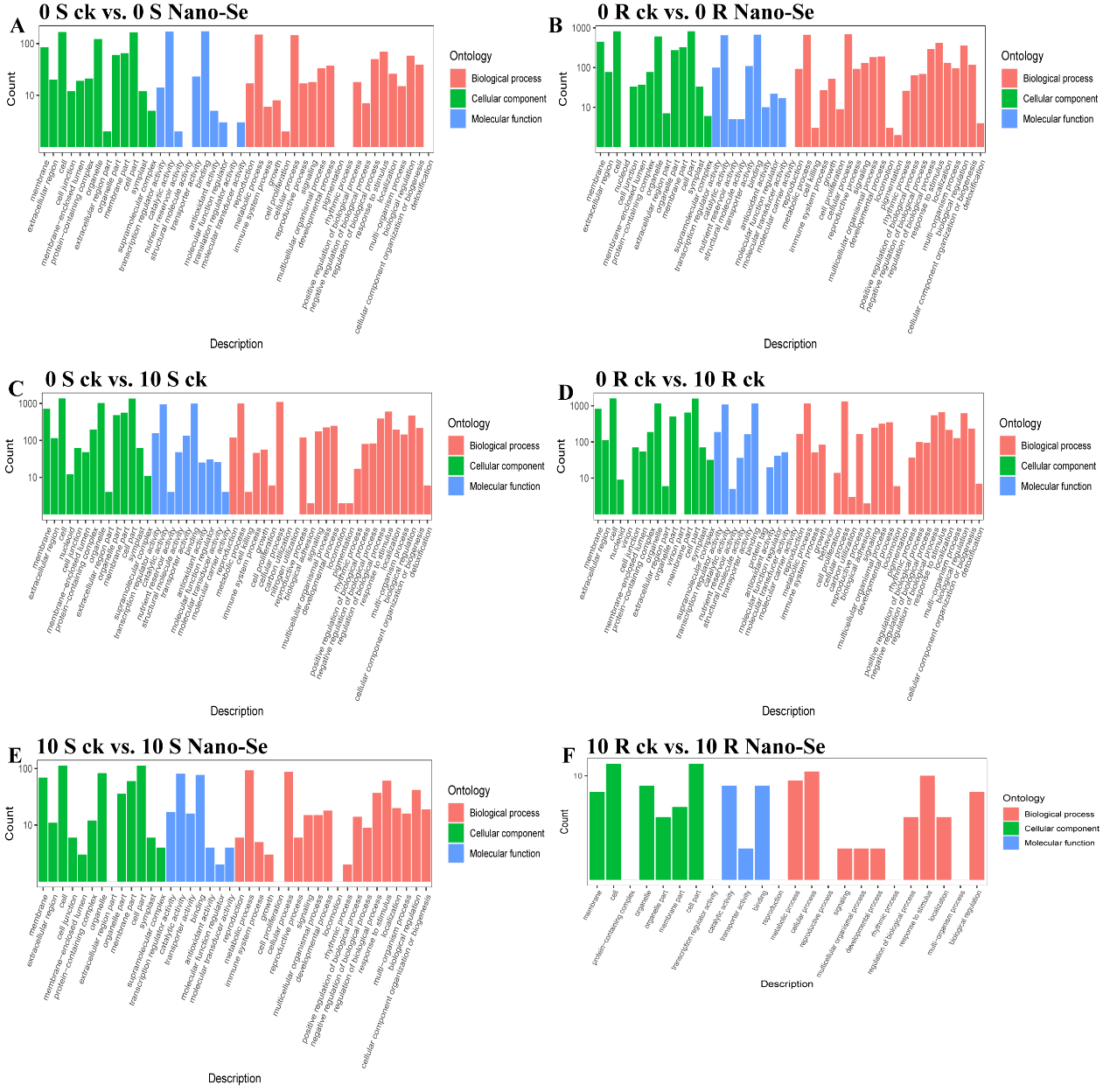
Fig. S9. GO enrichment analysis of leaf DEGs between susceptible and resistant cultivars and their response to powdery mildew infection with and without the Nano-Se pretreatment. Seedling leaves were used in the comparisons. A: 0 S ck *vs* 0 S Nano-Se, B: 0 R ck *vs* 0 R Nano-Se, C: 0 S ck *vs* 0 S ck, D: 0 R ck *vs* 10 R ck, E: 10 S ck *vs* 10 S Nano-Se, F: 10 R ck *vs* 10 R Nano-Se.


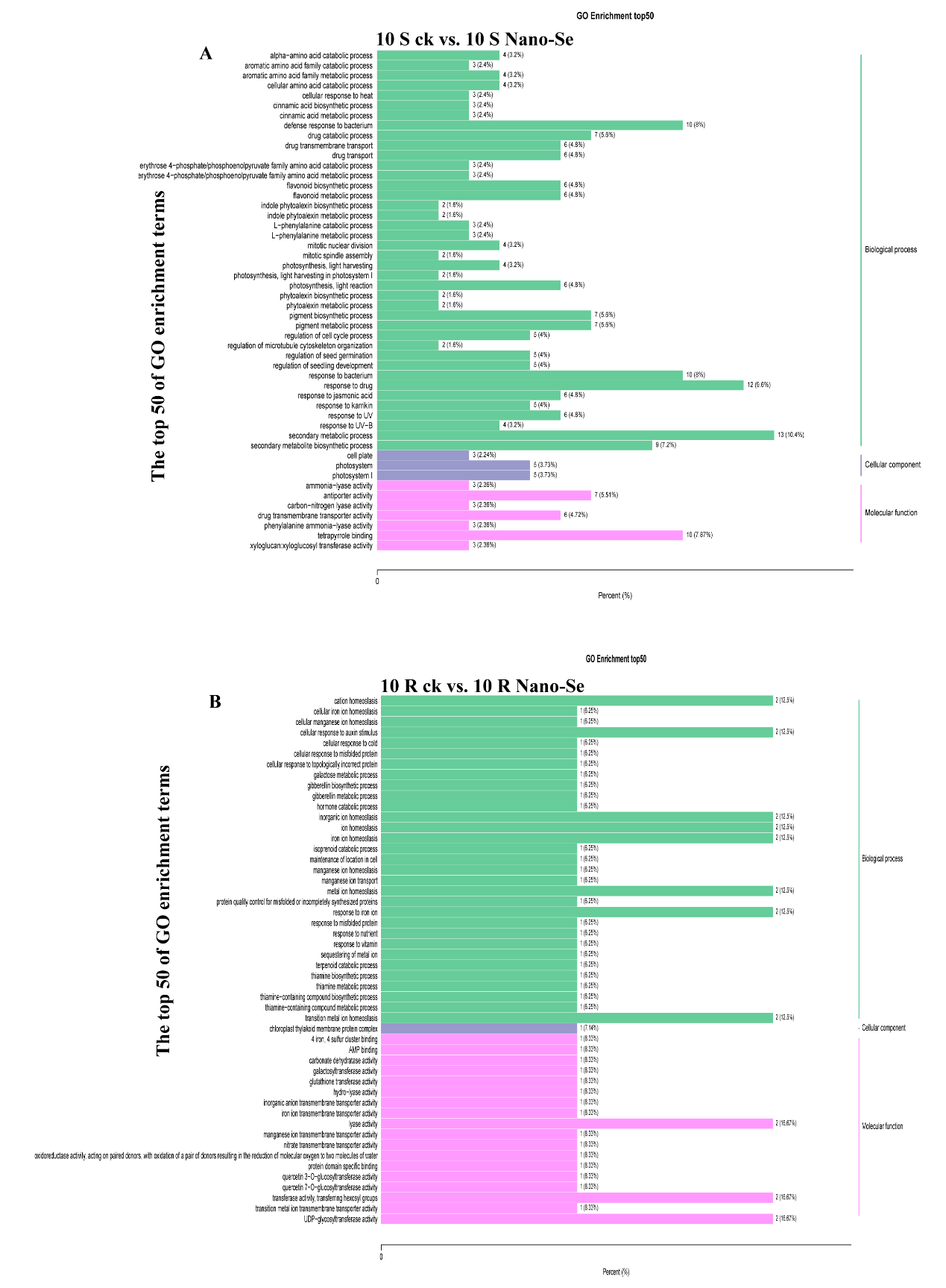


# Fig. S10. GO enrichment histogram of DEGs in response to the Nano-Se pretreatment in powdery mildew infected (10 dpi) leaves of the susceptible (S) and resistant (R) cultivars. The X-axis represents the ratio of the GO annotation of the DEGs to the total number GO annotation for all genes. The Y-axis represents the GO entry term. The label to the right of the graph represents the category to which the GO entry belongs. A: 10 S ck *vs* 10 S Nano-Se, B: 10 R ck *vs* 10 R Nano-Se.


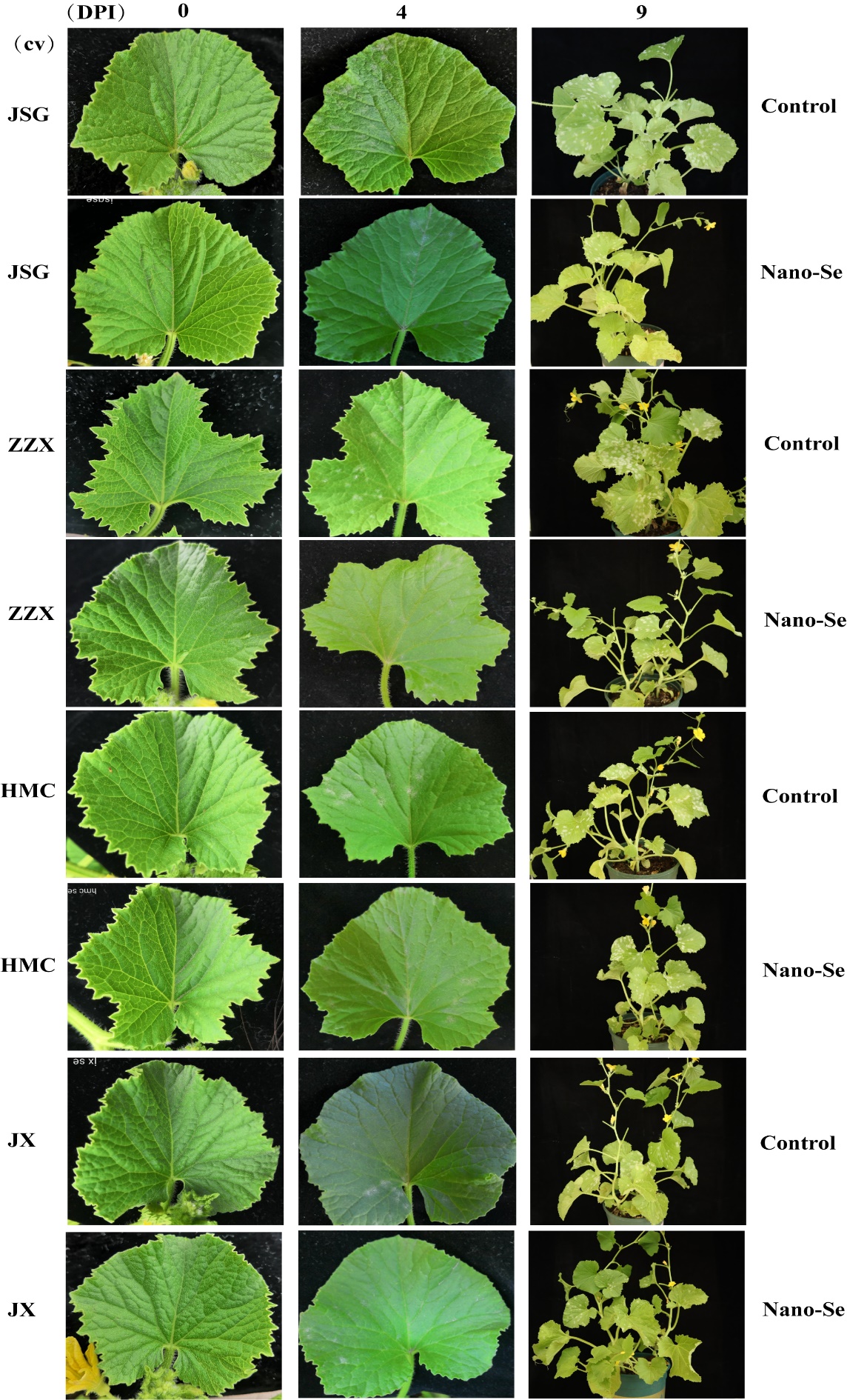


# Fig. S11. Melon seedlings of four different cultivars at different stages of treatment with Nano-Se. The days after infection (dpi) are indicted above the panels. The cultivars used (JSG, ZZX, HMC, and JX) are indicated to the left. The labels to the right indicate the rows of control and treated leaves (5.0 mg L^-1^), respectively.
